# Broad-Spectrum Virus Elimination by Nasal Mucosa-Colonized Wild-Type *Bacillus subtilis*

**DOI:** 10.34133/research.0781

**Published:** 2025-07-17

**Authors:** Yuchen Li, Chengjie Yang, Rongfeng Tang, Chengcheng Wang, Yunfeng Li, Wenwen Chao, Ahui Cui, Chun Liang, Ying Duan, Hui Zeng, Qian Yang

**Affiliations:** MOE Joint International Research Laboratory of Animal Health and Food Safety, College of Veterinary Medicine, Nanjing Agricultural University, Nanjing, Jiangsu 210095, PR China.

## Abstract

Outbreaks and the widespread prevalence of porcine respiratory infectious diseases have led to substantial economic losses worldwide. In this study, epidemiological surveillance revealed lower viral detection in association with an increased abundance of *Bacillaceae* in pigs with outdoor access; thus, *Bacillus subtilis* NS12, a strain with enhanced mucosal colonization and superior broad-spectrum antiviral activity, was isolated from the nasal mucosa of these pigs for further investigation. This mechanistic study revealed that the antiviral efficacy of *B. subtilis* NS12 is primarily attributed to bioactive metabolites, including a novel surfactin with high safety and antiviral activity and piceatannol, a potent antioxidant molecule. These metabolites modify the structure and fluidity of phospholipids within the viral envelope, thereby inhibiting viral entry by impeding membrane fusion. Therefore, with its broad-spectrum antiviral activity, *B. subtilis* NS12 offers a probiotic-based, environmentally sustainable, and noninvasive antiviral strategy for preventing and controlling respiratory viral infections.

## Introduction

Rapid expansion of the swine industry has boosted meat production efficiency; however, the risk of infectious diseases has increased. Research has shown that dense and confined farming systems increase pathogen transmission and mutation risks, threatening animal welfare and health [1,2]. Factors such as noise, inadequate ventilation, stringent rearing conditions, the routine administration of antibiotics, and standardized feed in intensive farming systems compromise the immune defenses of animals, thereby heightening their susceptibility to infections and contributing to increased disease prevalence [1,3]. In response, researchers and practitioners of animal husbandry are increasingly exploring alternative farming methodologies that emphasize natural conditions and prioritize animal welfare. Outdoor farming has received considerable attention in Europe, South Africa, and North America, with the potential of creating a natural living environment that promotes a wider array of innate behaviors in pigs [4–6]. This farming system involves outdoor activities that encourage pigs to interact with the land and vegetation while maintaining strict biosecurity. Studies have demonstrated that outdoor rearing considerably improves animal welfare and enhances their resistance to infectious diseases, particularly respiratory diseases. An epidemiological study involving 74 farms across 8 European Union countries found a marked lower incidence of respiratory infections in pigs that were allowed continuous outdoor access throughout their life cycle as compared to those raised exclusively indoors [7]. Pathological examinations of slaughterhouses in countries such as Denmark and Sweden have indicated that the prevalence of respiratory infectious diseases is considerably lower in outdoor-reared pigs than in pigs kept in confinement [8,9].

Research has demonstrated that the mucosal microbiomes of pigs raised outdoors, which are exposed to a wider variety of environmental microorganisms, exhibit markedly greater diversity and richness than those raised in confinement [10,11]. Mucosal surfaces in animals and humans, including the skin, gut, nasal cavity, and reproductive tract, host trillions of symbiotic microorganisms that are essential for biological defense, immune balance, and the maintenance of the mucosal barrier integrity [12–14]. More than 100 species of protective probiotics have been successfully identified in the gut, including *Bifidobacteria*, *Lactobacilli*, and *Akkermansia* [14]. These probiotics prevent pathogen colonization by competitively excluding harmful microbes and supporting mucosal barrier function through cellular components, such as the cell wall structure, surface proteins, and polysaccharides, as well as by secreting metabolites such as short-chain fatty acids (e.g., acetate, propionate, and butyrate) [15]. Consequently, the increased diversity of the respiratory mucosal microbiota observed in pigs reared outdoors may be a pivotal factor in the substantial enhancement of their mucosal immune function, facilitating effective resistance to respiratory infection.

The nasal cavity, being exposed to the external environment, serves as a primary point of entry for respiratory pathogens, and the mucosal microbiota helps prevent their spread to the lower respiratory tract [16,17]. Unlike the gut community, which stabilizes soon after birth, the respiratory microbiome matures gradually and remains highly sensitive to environmental cues [18], offering a tractable target for probiotic intervention. Emerging research highlights the role of nasal probiotics like *Staphylococcus epidermidis*, *Streptococcus thermophilus*, and certain *Bacillus* species in safeguarding the respiratory mucosa from infections [19–21]. However, our knowledge of how these nasal microbes function, especially in comparison to gut microbiota, is still developing, particularly regarding the identification of specific bacteria and their protective mechanisms. Furthermore, given the structural and physiological disparities between the nasal cavity and gut, a single probiotic may deploy distinct defenses in each niche*. Lactobacillus casei*, for example, survives the oxygen-rich airway by secreting hydrogen peroxide, which suppresses *Staphylococcus aureus*, *Haemophilus influenzae*, and *Moraxella catarrhalis* [22]. Further research into the roles and mechanisms of nasal probiotics is essential for developing strategies to improve respiratory health and fight infections.

Viral respiratory infections caused by pathogens such as the porcine reproductive and respiratory syndrome virus (PRRSV), swine influenza virus (SIV), and pseudorabies virus (PRV) pose serious challenges to large-scale intensive swine production systems. These pathogens are transmitted through airborne droplets and aerosols, triggering explosive outbreaks in pig populations [23]. Notably, a previous study revealed that the porcine epidemic diarrhea virus (PEDV), traditionally classified as an enteric pathogen, can also be transmitted via the air and infiltrate piglets through the nasal mucosa, leading to severe intestinal disease [24]. Vaccine-based control, although indispensable, is hindered by lengthy strain-matching cycles, limited serotype coverage, and rapidly waning efficacy in the face of frequent antigenic drift and recombination, thereby exacerbating the complexity and severity of respiratory infections in modern swine units. To address these challenges, broad-spectrum and efficient antiviral strategies are required that can safeguard the health of pigs and promote sustainable development in the swine industry.

In this study, we observed that outdoor-reared pigs carry markedly lower nasal viral loads than their indoor-reared counterparts, indicating that husbandry practices shape susceptibility to respiratory infection. Epidemiological surveillance, 16*S* ribosomal RNA (rRNA) profiling, and statistical modeling identified colonization by *Bacillus subtilis* as the principal protective factor. We isolated a wild-type strain, NS12, from outdoor pig nasal swabs and demonstrated its stable persistence in the nasal mucosa. We showed that its secreted surfactin together with the stilbenoid piceatannol cooperatively inactivated a broad panel of enveloped viruses. To date, probiotic research has centered chiefly on gut-derived isolates and their immunomodulatory effects, leaving the defensive strategies of nasal commensals—especially metabolite-mediated mucosal protection—largely unexplored. Our study closes this gap by identifying an ecology-matched nasal probiotic, *B. subtilis* NS12, and revealing a metabolite-driven, envelope-disruptive mechanism that underlies its broad-spectrum antiviral activity. These insights refine the recognized role of *B. subtilis* in animal health and lay a theoretical foundation for eco-friendly, effective mucosal antivirals, opening a new avenue for the systematic control of swine respiratory diseases.

## Results

### *B. subtilis* colonization lowers nasal virus detection in pigs with outdoor access

Surveillance analysis of the nasal pathogens in swine raised in complete confinement and in those with outside access demonstrated markedly lower viral detection in pigs raised under outdoor conditions (Fig. S1A). As shown in Fig. S1B, 86.5% of the nasal mucosa samples from outdoor-accessing pigs tested negative for pathogens, whereas 67.6% of the indoor-raised pigs tested positive for one or more pathogens, with 7.2% of the weaned piglets carrying up to 3 distinct viruses. Detection rates of 41% and 36% were obtained for PRRSV and SIV in the nasal mucosa of indoor-raised pigs, respectively, with significantly lower levels observed in outdoor-accessing pigs, suggesting these viruses as the predominant respiratory pathogens in confined settings (Fig. S1C). Although PEDV primarily affects the gastrointestinal tract, its positivity rate in nasal swabs from indoor-raised pigs reached 23%, with significant reduction in outdoor-accessing pigs (Fig. S1C).

To elucidate the mechanisms underlying the reduced pathogen detection rate in outdoor-accessing pigs, a comprehensive analysis of the nasal microbiota of indoor and outdoor pigs was conducted using 16*S* rRNA gene sequencing. The results were evaluated using the abundance-based coverage estimator (ACE) and Simpson indices and indicated that outdoor rearing significantly enhances both the taxon abundance and diversity of the nasal microbiome compared to confined rearing (Fig. S2A and B). Principal components analysis (PCA) further demonstrated distinct differences in the composition and structure of the nasal microbiome for the 2 rearing systems (Fig. S2C), with taxonomic-level changes indicating notably increased abundances of *Firmicutes*, *Actinobacteria*, and *Tenericutes* and reduced levels of *Bacteroidetes* and *Proteobacteria* in outdoor-accessing pigs (Fig. S2D). The marked increase in *Firmicutes*, which are predominantly beneficial, prompted further investigation of the specific communities within this phylum, which revealed a significant increase in the abundance of *Bacillus* in the nasal cavity of pigs with outdoor access (Fig. S2E). Notable increases in both *Lactobacillales* and *Bacillales* were also observed in outdoor-accessing pigs (Fig. S2F). Notably, 2 key probiotic families, *Bacillacea*e (*Bacillales*) and *Lactobacillaceae* (*Lactobacillales*), showed significant increases within the nasal cavities of pigs with outdoor access (Fig. S2G and H).

**Fig. 1.**
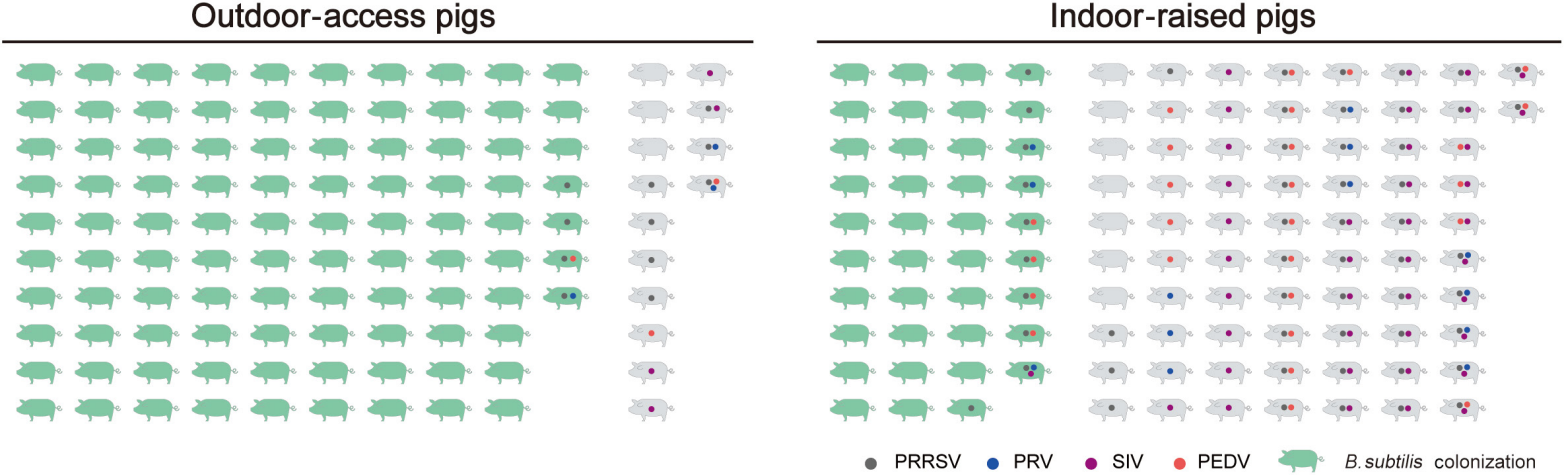
Correlations between decreased respiratory viral detection rate and presence of *Bacillus* in the nasal mucous of pigs exposed to different rearing systems. Porcine reproductive and respiratory syndrome virus (PRRSV), pseudorabies virus (PRV), swine influenza virus (SIV), and porcine epidemic diarrhea virus (PEDV) detection in nasal swabs from indoor-reared and outdoor-accessing pigs. Green pigs indicate the presence of *Bacillus* in the nasal cavity.

Real-time quantitative polymerase chain reaction (RT-qPCR) was utilized to confirm the distribution of *B. subtilis* in nasal swabs previously tested for pathogens, and the results revealed a significant inverse correlation between the presence of *B. subtilis* and viral detection in piglets (Fig. 1). As shown in Fig. S1D, pigs with outdoor access exhibited a significantly higher prevalence of *B. subtilis* than those raised indoors, with significantly lower viral detection rates observed in swabs containing *B. subtilis* (Fig. S1E). Specifically, in the group raised indoors, 90.3% of the nasal swabs that were devoid of *B. subtilis* tested positive for viral presence, with detection rates of 31.9%, 48.6%, and 9.7% obtained for 1, 2, and 3 viral types, respectively. In contrast, a significantly reduced viral detection rate of 25.6% was observed for swabs containing *B. subtilis*. In the group with outdoor access, 78.6% of the nasal swabs with no *B. subtilis* tested positive for viruses, with detection rates of 57.1%, 14.3%, and 7.1% obtained for 1, 2, and 3 viral types, respectively. Again, significantly reduced viral detection was observed for samples containing *B. subtilis*, of which 95.9% tested negative for viruses. These findings suggest that colonization with *B. subtilis* may be a key protective factor contributing to the lower pathogen detection rate in the nasal mucosa of pigs with outdoor access.

### Broad-spectrum antiviral activity of *B. subtilis* NS12 isolated from pigs with outdoor access

A total of 14 *B. subtilis* strains were isolated from the nasal cavities of outdoor-accessing pigs, with isolates consistently producing large, circular, oval, off-white, wrinkled colonies on Luria–Bertani (LB) agar plates. The isolates were identified as gram-positive rods exhibiting the characteristic morphological features of *B. subtilis* (Fig. S3A and B). Positive results for the housekeeping gene *gyrA* confirmed the presence of *B. subtilis* (Fig. S3C). Malachite green staining indicated that all 14 strains could produce endospores, consistent with known *B. subtilis* characteristics (Fig. S3D). Biochemical tests further supported the *Bacillus* classification, with positive results obtained from glucose fermentation, starch hydrolysis, oxidase, and Voges–Proskauer tests, and negative results from gas production and gelatin liquefaction tests. To elucidate the taxonomic position of wild-type *B. subtilis* strains, 16*S* ribosomal DNA (rDNA) sequencing and phylogenetic analysis were conducted, with isolates showing sequence similarities of 96.1% to 99.6% with the reference *B. subtilis* strain (Fig. S3E). Phylogenetic analysis based on 16*S* rRNA sequences revealed that the wild-type *B. subtilis* isolates were located in several clades of classic *B. subtilis* strains, with strain NS12 clustering together with *B. subtilis* SBMP4 (Fig. S3F).

Recombinant PEDV expressing green fluorescent protein (GFP), designated PEDV-GFP, was used to assess the antiviral efficacy of the various *B. subtilis* isolates through 2 distinct approaches: pretreatment of the host cells and pretreatment of the virus. The standard *B. subtilis* strain 168, which lacks antiviral activity, was used as a control (Fig. S4A). The results demonstrated that pretreatment of host cells with the isolates did not appreciably alter GFP expression in PEDV-infected cells (Fig. S4B); however, pretreatment of the virus with isolates NS3, NS7, NS10, NS11, NS12, and NS13 led to a marked reduction in GFP expression (inhibition rate >60%) in Vero E6 cells, demonstrating the potent antiviral activity (Fig. S4C). To rule out the possibility that the observed antiviral effects were due to cytotoxicity, the cytotoxicities of the *B. subtilis* isolates NS3, NS7, NS10, NS11, NS12, and NS13 were evaluated in Vero E6, PK15, and Marc145 cells using the CC8 assay, with the non-antiviral isolate NS1 as a negative control. The results showed that none of the tested *B. subtilis* isolates were cytotoxic on the 3 cell lines at concentrations ranging from 10^6^ to 10^8^ colony-forming units (CFU) (Fig. S5).

**Fig. 2.**
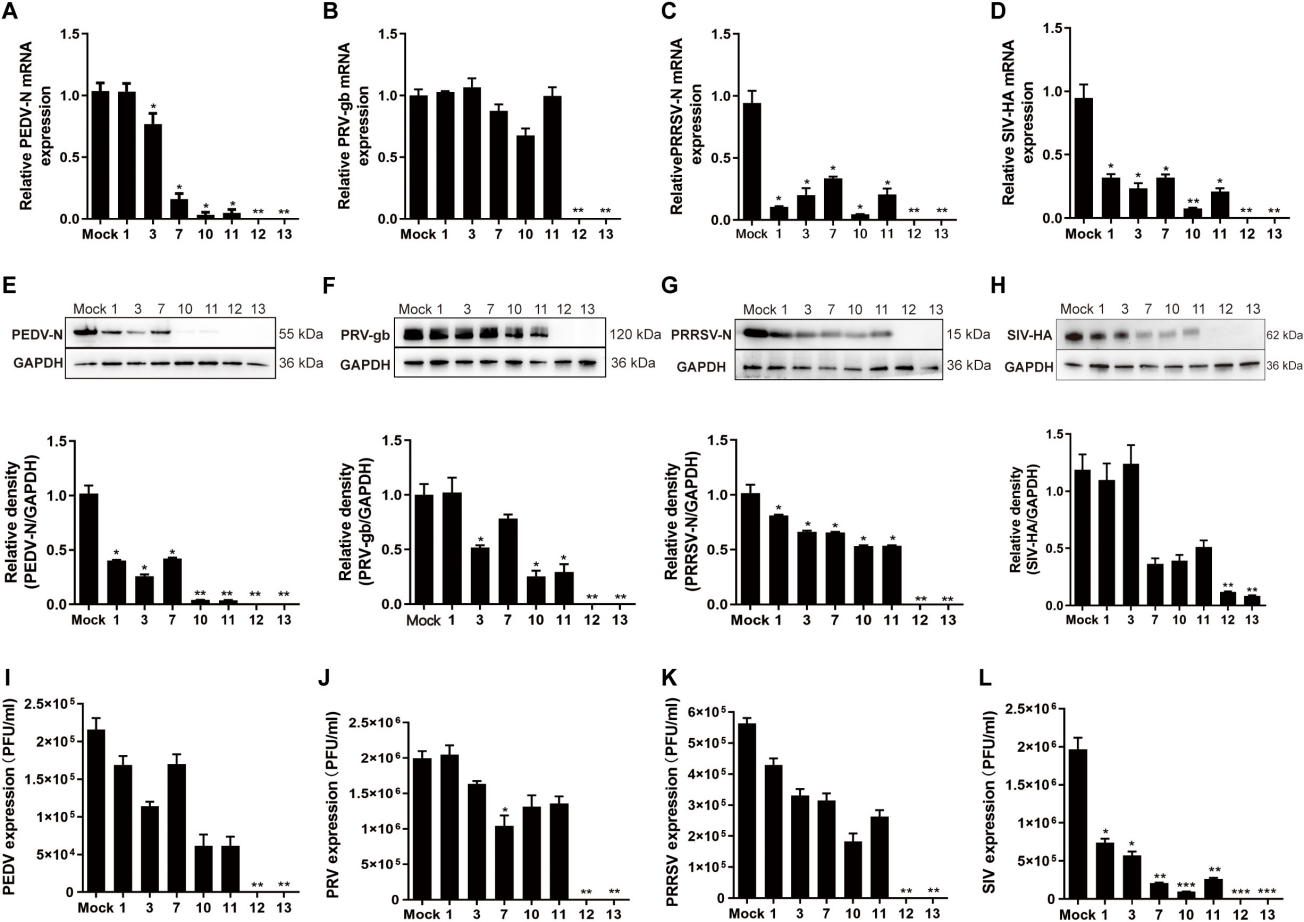
Evaluation of the antiviral effects of *B. subtilis* candidates against common porcine viral pathogens. Inhibitory effects of preincubating *B. subtilis* isolates NS1, NS3, NS7, NS10, NS11, NS12, and NS13 on PEDV, PRV, and PRRSV infection. (A to D) Viral mRNA levels in cells, including the N gene in PEDV (A), gB gene in PRV (B), N gene in PRRSV (C), and HA gene in SIV (D) determined using RT-qPCR. (E to H) Viral protein expression in cells assessed via Western blotting, including the N protein in PEDV (E), gB protein in PRV (F), N protein in PRRSV (G), and HA protein in SIV (H). Bar graphs show relative protein expression levels from Western blot results. (I to L) Plaque formation assays of infectious viral particles in cell supernatants, with bar graphs summarizing the results for PEDV (I), PRV (J), PRRSV (K), and SIV (L). All data are presented as means ± SD, and comparisons were performed using one-way analysis of variance (ANOVA). *P < 0.05, **P < 0.01. Results were obtained from at least 3 independent experiments.

To assess the broad-spectrum antiviral activity of the candidate *B. subtilis* strains, isolates were incubated with PEDV, PRV, and PRRSV and then inoculated into their respective host cells. Viral replication evaluation after 24 h showed that *B. subtilis* strains NS12 and NS13 exhibited the strongest antiviral activity against all 3 viruses as compared with other isolates. RT-qPCR and Western blot analysis demonstrated the complete inhibition of viral gene and protein expression of PEDV (Fig. 2A and E), PRV (Fig. 2B and F), PRRSV (Fig. 2C and G), and SIV (Fig. 2D and H), while plaque assays verified the total suppression of viral titers in cells treated with NS12 and NS13 (Fig. 2I to L). The adhesive properties of the *B. subtilis* isolates were further evaluated using porcine respiratory epithelial cells. Notably, isolate NS12 exhibited significantly enhanced adherence to swine nasal epithelial cells compared to NS13 (Fig. S6A and B), and in vivo studies indicated the persistence of *B. subtilis* NS12 in the nasal cavity of piglets or approximately 1 to 2 weeks following intranasal administration, indicating its capacity for mucosal colonization (Fig. S6C and D). Based on these findings, *B. subtilis* NS12 was selected for further investigation.

### *B. subtilis* NS12 enhances host mucosal defense against various viral infections

**Fig. 3.**
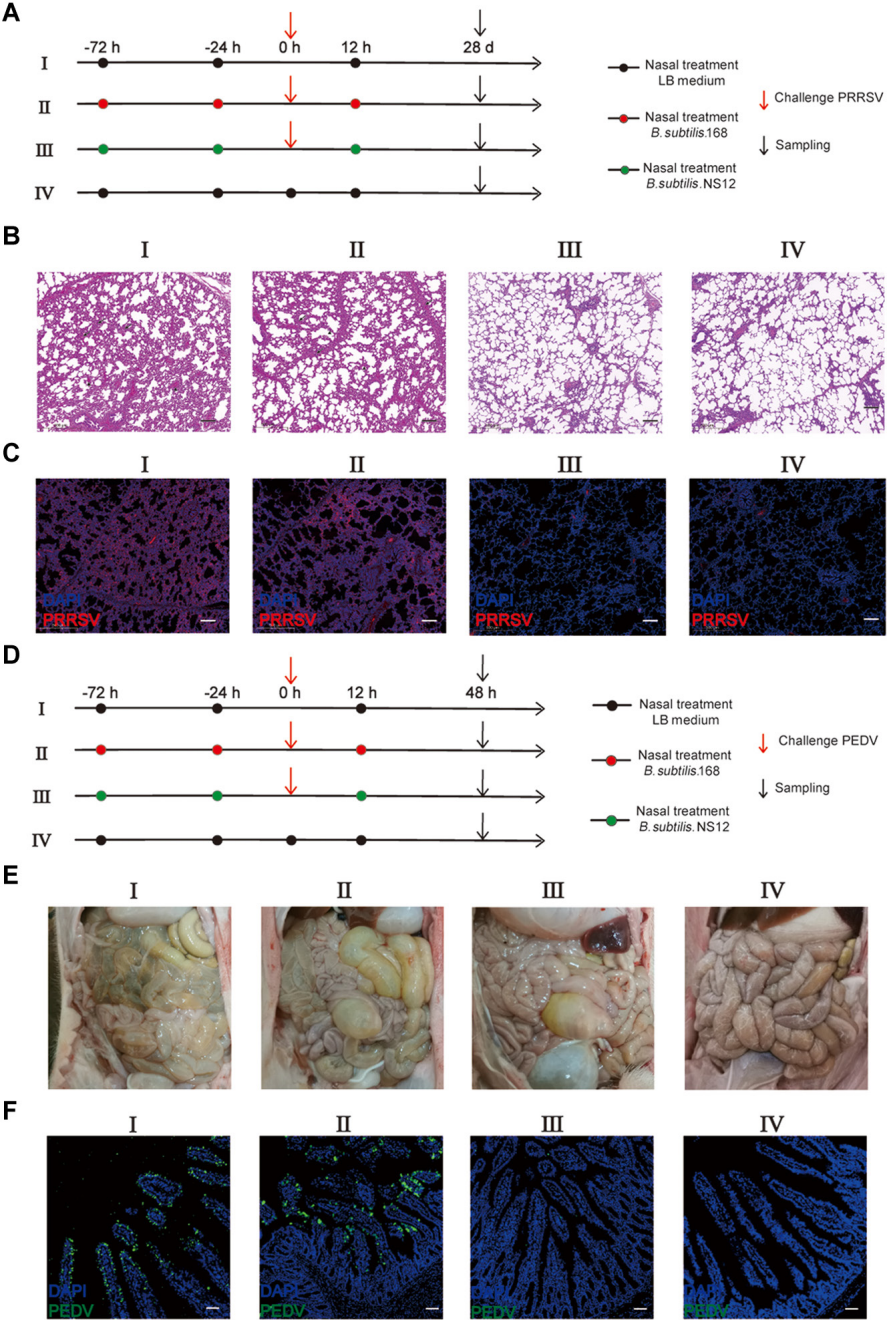
*B. subtilis* NS12 provides protection against PRRSV and PEDV nasal infection in pigs. (A) Schematic of the PRRSV nasal challenge experiment to assess the protective efficacy of *B. subtilis* NS12 in piglets (*n* = 5). Experimental groups included the PRRSV infection group (I), *B. subtilis* 168 nasal administration group (II), *B. subtilis* NS12 nasal administration group (III), and blank control group (IV). (B) Histopathological examination of lung tissue from each group. An arrow indicates thickening of the interlobular septa around the alveoli, and a triangle indicates the presence of inflammatory cells, necrotic debris, and exfoliated epithelial cells infiltrating the bronchioles. (C) Distribution of PRRSV in lung tissues, as detected by IF staining with an anti-PRRSV N monoclonal antibody (red) (scale bar, 20 μm). (D) Schematic diagram of the PEDV nasal challenge experiment to evaluate the protective effect of *B. subtilis* NS12 in piglets. Experimental groups comprised a PEDV infection group (I), *B. subtilis* 168 nasal administration group (II), *B. subtilis* NS12 nasal administration group (III), and blank control group (IV). (E) Gross pathological examination of small intestines from each group. (F) Distribution of PEDV in intestinal tissues from each group, determined by IF staining with an anti-PEDV monoclonal antibody (green). Nuclei were counterstained with DAPI (blue) (scale bar, 20 μm). All data are presented as means ± SD, with comparisons performed using one-way ANOVA. *P < 0.05, **P < 0.01.

To further verify the in vivo antiviral efficacy of *B. subtilis* NS12, its protective effects against PRRSV infection were evaluated in 1-month-old specific pathogen-free (SPF) piglets (Fig. 3A). Piglets in both group I (LB medium) and group II (*B. subtilis* 168) exhibited a significant rise in body temperature of above 41 °C upon infection, which persisted 4 days after infection. In contrast, a mild temperature increase was observed in the NS12-treated group (group III) (Fig. S7A). Quantification of the serum viral load by RT-qPCR on days 3, 7, and 10 post-infection indicated significantly lower serum viral loads in the NS12-treated group compared to those in groups I and II, as seen in Fig. S7B. Necropsies were performed and lung samples were collected on day 10 for pathological examination, with results revealing pronounced pulmonary lesions in piglets from groups I and II, including thickened alveolar septa, hemorrhage, and the infiltration of inflammatory cells (Fig. 3B). Treatment with *B. subtilis* NS12 substantially mitigated these pathological changes, with immunofluorescence (IF) and RT-qPCR results indicating significantly reduced viral loads in the lungs of the NS12-treated group compared to those in both the LB control group and the *B. subtilis* 168 control group (Fig. 3C and Fig. S7C).

The protective efficacy of nasal inoculation with *B. subtilis* NS12 against PEDV infection was also evaluated (Fig. 3D). Within 60 h of infection, piglets in the LB medium control group (group I) and the *B. subtilis* 168 control group (group II) exhibited typical symptoms of PEDV infection, with acute watery diarrhea, vomiting, and yellow malodorous stools around the perianal region. Necropsy revealed thin and transparent intestinal walls. In contrast, no observable pathological changes were detected in piglets that received nasal NS12 administration (group III) (Fig. 3E). IF analysis revealed numerous PEDV-infected epithelial cells in the small intestines of piglets from groups I and II, whereas viral proteins were undetectable in the small intestines of piglets in the NS12-treated group (Fig. 3F). Furthermore, RT-qPCR and Western blot analyses showed significantly lower PEDV transcription and protein expression in group III than in groups I and II (Fig. S7D and E). Considering previous reports of *B. subtilis* germination in the gastrointestinal tract, the protective effects of orally administered NS12 were further examined (Fig. S8A), with piglets in groups I and II exhibiting typical symptoms and intestinal changes 48 h post-PEDV oral infection, while neither symptoms nor pathological changes were observed in the NS12-treated group (Fig. S8B). IF, RT-qPCR, and Western blot results further confirmed a significant reduction in the viral load of pigs in the NS12 group compared to those in groups I and II (Fig. S8C to E).

The protective efficacy of *B. subtilis* NS12 against PRV infection was also evaluated in a mouse model (Fig. S9A). As shown in Fig. S9B, groups that were pretreated or treated with *B. subtilis* 168 exhibited mortality from day 3 post-infection with PRV, with 100% mortality observed by day 5. In contrast, only 2 of the 6 mice in the NS12 treatment group died by day 4, with a survival rate of 66.7%, and all mice in the NS12 pretreatment group survived the challenge (Fig. S9B). Necropsies on 5 mice from each group 60 h post-infection revealed severe brain and lung hemorrhage and pronounced inflammation in the PRV and *B. subtilis* 168 groups, with the NS12 treatment group exhibiting markedly reduced tissue damage and the NS12 pretreatment group demonstrating a complete absence of pathological changes (Fig. S9C and D). RT-qPCR analysis (Fig. S9E) indicated a markedly lower viral load in the brain and lung tissues of both the NS12 treatment and NS12 pretreatment groups. These findings strongly indicate that nasal inoculation with *B. subtilis* NS12 enhances host resistance to viral infection.

### *B. subtilis* NS12 exerts direct antiviral effects by secreting highly stable metabolites

**Fig. 4.**
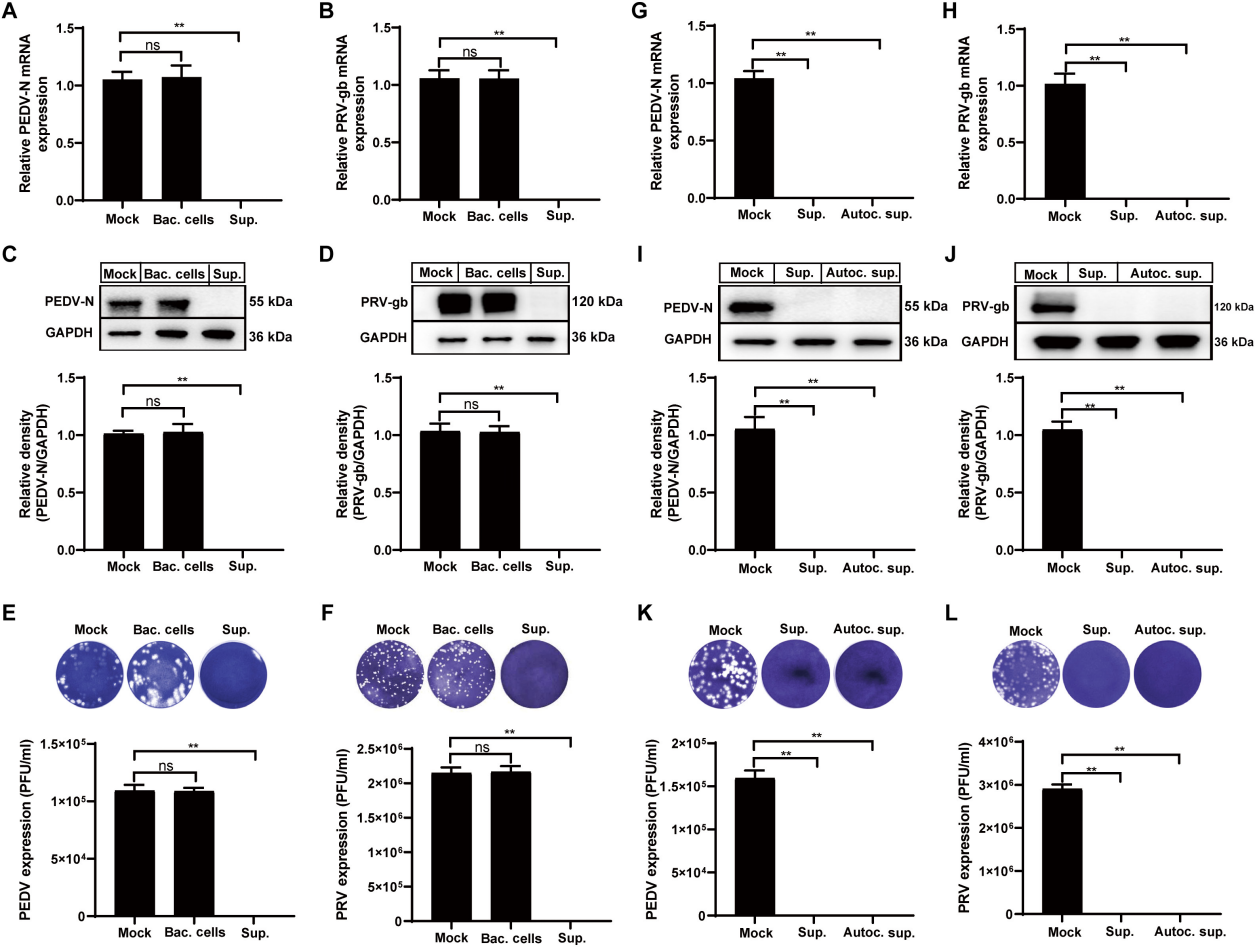
Characterization of the antiviral component of *B. subtilis* NS12. (A to F) Cell pellets and culture supernatants of NS12 incubated with PEDV and PRV, followed by inoculation in Vero E6 cells. Transcription of PEDV N (A) and PRV gB (B) genes was assessed by qRT-PCR. Protein expression of PEDV N (C) and PRV gB (D) was analyzed by Western blot. Plaque assays measured infectious viral particles of PEDV (E) and PRV (F) in the supernatants. (G to L) Cultured NS12 supernatants were subjected to autoclaving, followed by evaluation of antiviral activity. Transcription of PEDV N (G) and PRV gB (H) genes as assessed by qRT-PCR. Protein levels of PEDV N (I) and PRV gB (J) analyzed by Western blot. Plaque assays were used to determine viral titers of PEDV (K) and PRV (L) in the supernatants. Data are presented as means ± SD from 3 independent experiments. Statistical significance was determined using one-way ANOVA; ns, not significant; *P < 0.05; **P < 0.01.

Due to the broad-spectrum antiviral properties of *B. subtilis* NS12 and its mechanism of directly interacting with viruses, it is likely that its antiviral mechanisms share similarities across different viral types. To further explore the specific antiviral mechanisms of *B. subtilis* NS12, we selected 2 representative viral models: PRV, which can infect the host through multiple routes (with the nasal mucosa being considered one of its primary entry points), and PEDV, an enteric virus that primarily infects through the gastrointestinal tract. To identify the specific component that is responsible for the antiviral activity of *B. subtilis* NS12, both bacterial cells and culture supernatant were evaluated, with results showing no significant effect on viral infectivity for the bacterial cells and pronounced antiviral properties for the supernatant. Notably, pretreatment with the NS12 supernatant significantly reduced viral mRNA (Fig. 4A and B), protein expression (Fig. 4C and D), and the production of progeny virions (Fig. 4E and F) in both PEDV-infected Vero E6 cells and PRV-infected PK15 cells. To determine whether the observed antiviral activity could be attributed to the proteins present in the supernatant, the supernatant was autoclaved and its heat stability was assessed. Remarkably, the antiviral efficacy of the supernatant remained intact after autoclaving, with significantly reduced viral gene transcription (Fig. 4G and H), protein synthesis (Fig. 4I and J), and infectious particle release (Fig. 4K and L). These findings suggest that the antiviral activity of *B. subtilis* NS12 is mediated by its secreted supernatant, with the active compounds exhibiting substantial heat resistance, underscoring their potential stability and efficacy.

### *B. subtilis* NS12 produces surfactin molecules with enhanced safety and antiviral activity

*B. subtilis* is well known for synthesizing various lipopeptides, particularly surfactin, which is a cyclic lipopeptide biosurfactant. Surfactin consists of an anionic heptapeptide ring and hydrophobic β-hydroxy fatty acids with chain lengths ranging from 13 to 15 carbon atoms. This amphiphilic structure imparts pronounced stability to the molecule [25]. The antiviral properties of surfactin, specifically its ability to inhibit viral fusion with host cell membranes, have been previously confirmed by our group [26]. Therefore, we propose that the consistent antiviral activity observed in the *B. subtilis* NS12 supernatant may be attributed to the presence of surfactin.

**Fig. 5.**
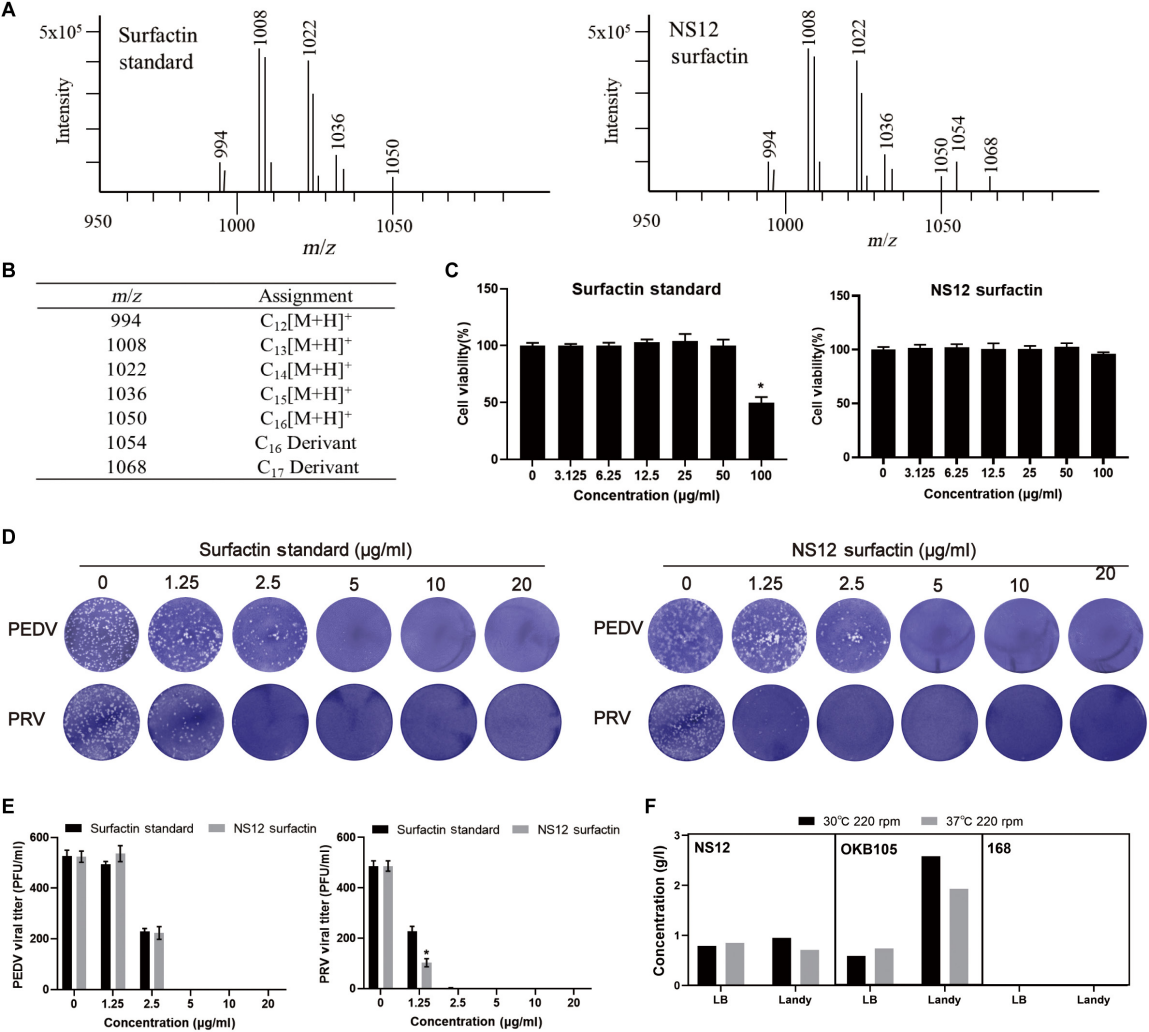
Antiviral activity and physicochemical properties of surfactin from *B. subtilis* NS12. (A) Mass spectra of surfactin (SF) standard (98% purity) and surfactin purified from *B. subtilis* NS12. (B) Assignment of surfactin homologs, with subscripts indicating the number of carbon atoms in the aliphatic chain. (C) Cytotoxicity of *B. subtilis* NS12 lipopeptides assessed using a CCK-8 assay, with standard lipopeptides as controls. (D and E) Antiviral effects of standard surfactin and surfactin extracted from *B. subtilis* NS12 against PEDV and PRV. The comparison was performed using plaque inhibition assays (D), with results given statistically and presented using bar graphs (F). Data are presented as means ± SD from 3 independent experiments. Statistical significance was determined using one-way ANOVA; ns, not significant; *P < 0.05; **P < 0.01.

Based on their chemical properties, surfactants were extracted from the supernatant of *B. subtilis* NS12 cultures for exploration. Liquid chromatography–tandem mass spectrometry (LC-MS/MS) confirmed the presence of surfactants, with ion peaks detected at mass-to-charge ratio (*m*/*z*) = 994, 1,008, 1,022, 1,036, and 1,050, consistent with standard surfactant profiles (Fig. 5A), as well as 2 novel surfactant homologs identified with *m*/*z* values of 1,054 and 1,068. Further MS analysis revealed that the ions at *m*/*z* = 1,050 and 1,054 shared similar N-terminal products, both of which contained fatty acid chains, indicating that the ions are derivatives formed by the loss of specific amino acid residues (such as Leu, Leu-Asp, or Leu-Asp-Val). The molecular weight difference of 4 Da between the *m*/*z* = 1,050 and 1,054 ions can be attributed to the molecular weight difference between carbon (C) and oxygen (O) and suggests the involvement of an oxygen atom at *m*/*z* = 1,054 or the removal of a carbon atom relative to its precursor. Moreover, the observed molecular weight difference of 14 Da between *m*/*z* = 1,054 and *m*/*z* = 1,068 indicates that the latter may possess a longer side chain, specifically -CH2. These findings support the hypothesis that *m*/*z* = 1,054 and 1,068 represent C16 and C17 surfactant derivatives, respectively (Fig. 5B).

The NS12 surfactin extract showed minimal cytotoxicity at 100 μg/ml in the Cell Counting Kit-8 (CCK-8) assay, with almost 100% cell viability, while standard surfactin reduced the viability to approximately 50% (Fig. 5C). This suggests a considerably better safety profile for NS12 surfactin extract compared to that for the standard. The antiviral activity of NS12-derived surfactin was assessed at safe concentrations, with plaque reduction assays indicating strong antiviral effects for NS12 surfactin, similar to those of the commercial standard, but with markedly better PRV replication inhibition (Fig. 5D and E). Moreover, *B. subtilis* NS12 exhibited a robust capacity for surfactin synthesis under standard laboratory conditions (37 °C, 220 rpm in LB medium), with a yield of 0.845 g/l (Fig. 5E). This substantial yield was obtained without the need for specialized media, such as Landy medium, which is conventionally used to enhance lipopeptide production [27]. In comparison, the reference strain *B. subtilis* 168 produced negligible amounts of surfactin under identical conditions. The engineered recombinant strain *B. subtilis* OKB105 produced a significantly higher yield of 2.58 g/l under optimal conditions (30 °C in Landy medium); however, under identical conditions to those used for NS12 (37 °C, 220 rpm in LB medium), the yield was limited to 0.74 g/l (Fig. 5F).

### *B. subtilis* NS12 exerts direct antiviral effects by producing the phenolic compound piceatannol

**Fig. 6.**
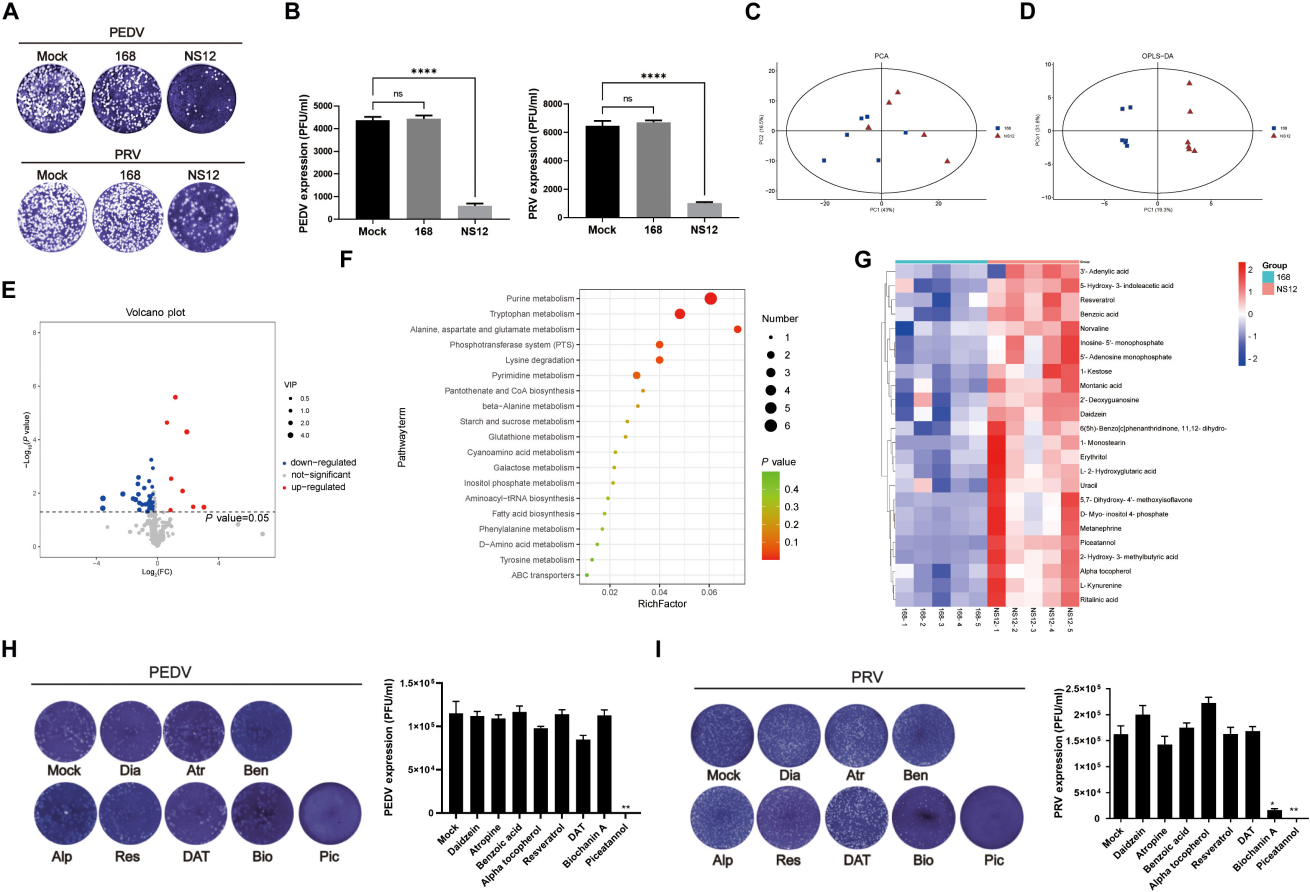
Screening and identification of antiviral small-molecule metabolites in culture supernatant of *B. subtilis* NS12. (A and B) A plaque inhibition assay was used to evaluate the antiviral activity of *B. subtilis* NS12 and standard strain 168 (A). Plaque results are given statistically and presented as bar graphs (B). (C to F) Untargeted LC-MS metabolomics analysis of culture supernatant from *B. subtilis* NS12, with supernatant from *B. subtilis* 168 (which lacks antiviral activity) as a control. (C and D) PCA and OPLS-DA analysis revealed clearly distinct metabolomic profiles between the 2 *B. subtilis* strains. (E) Volcano plot of differentially expressed metabolites in the culture supernatants of the 2 *B. subtilis* strains. (F) Bubble plot showing the top 20 enriched metabolic pathways. (G) Hierarchical clustering heatmap of differential metabolites. (H and I) Antiviral effects of candidate metabolites on PEDV (G) and PRV (I) infection detected using a plaque inhibition assay. Data are presented as means ± SD from 3 independent experiments. Statistical significance was determined using one-way ANOVA; ns, not significant; *P < 0.05; **P < 0.01. PCA, principal components analysis; OPLS-DA, orthogonal partial least squares-discriminant analysis; Dia, daidzein; Atr, atropine; Ben, benzoic acid; Alp, α-tocopherol; Res, resveratrol; HMBA, 2-hydroxy-3-methylbutyric acid; Bio, biochanin A; Pic, piceatannol.

Following the acid precipitation of surfactin, the supernatant derived from *B. subtilis* NS12 was collected and adjusted to a neutral pH. Notably, plaque inhibition assays demonstrated that the neutralized supernatant retained substantial antiviral activity, suggesting additional antiviral compounds in the secretome of *B. subtilis* NS12 and highlighting the need for further investigation to identify these compounds (Fig. 6A). To explore the specialized metabolites associated with antiviral activity, a nontargeted metabolomic approach was used to analyze the culture supernatant of *B. subtilis* NS12, with PCA and orthogonal partial least squares discriminant analysis (OPLS-DA) revealing distinct clustering patterns for the *B. subtilis* 168 and NS12 strains, with clear separation in their metabolic profiles despite partial overlap (Fig. 6B and C). Volcano plot analysis identified 47 differentially expressed metabolites, with 8 up-regulated in strain 168 and 39 up-regulated in NS12 (Fig. 6D). Enrichment analysis showed that *B. subtilis* NS12 is rich in purine, tryptophan, alanine, aspartate, glutamate metabolism, the phosphotransferase system, and lysine degradation pathways, which are vital for nucleotide synthesis, amino acid metabolism, energy production, enhanced growth, adaptation, and survival (Fig. 6E).

A comprehensive literature review identified 8 metabolites with potential antiviral properties among the 39 up-regulated metabolites secreted by *B. subtilis* NS12, including daidzein, atropine, benzoic acid, α-tocopherol, resveratrol, 2-hydroxy-3-methylbutyric acid, biochanin A, and piceatannol (Fig. 6F). The optimal concentrations of these compounds were determined based on previous studies and the results of a CCK-8 assay (Fig. S10). Plaque inhibition assays were conducted to evaluate the inhibitory effects of the compounds on PEDV and PRV, with the results indicating that biochanin A notably inhibits PRV infection, whereas piceatannol demonstrated even more potent antiviral activity against both PEDV and PRV, highlighting its broad-spectrum antiviral potential. Notably, resveratrol, also a member of the polyhydroxylated stilbene class and a hydroxylated analog of piceatannol that lacks a hydroxyl group at the 4′ position, exhibited no appreciable antiviral effect (Fig. 6G and H). Further antiviral activity assays demonstrated strong efficacy for piceatannol against both PRV and PEDV, with half-maximal effective concentrations (EC_50_) of less than 10 μM (Fig. S11A and B). Furthermore, piceatannol effectively inhibited PEDV after only 10 min of incubation, whereas PRV was entirely suppressed immediately after mixing, eliminating the need for further incubation (Fig. S11C). Notably, piceatannol retained robust antiviral activity against PEDV and PRV even at 4 °C (Fig. S11D and E).

**Fig. 7.**
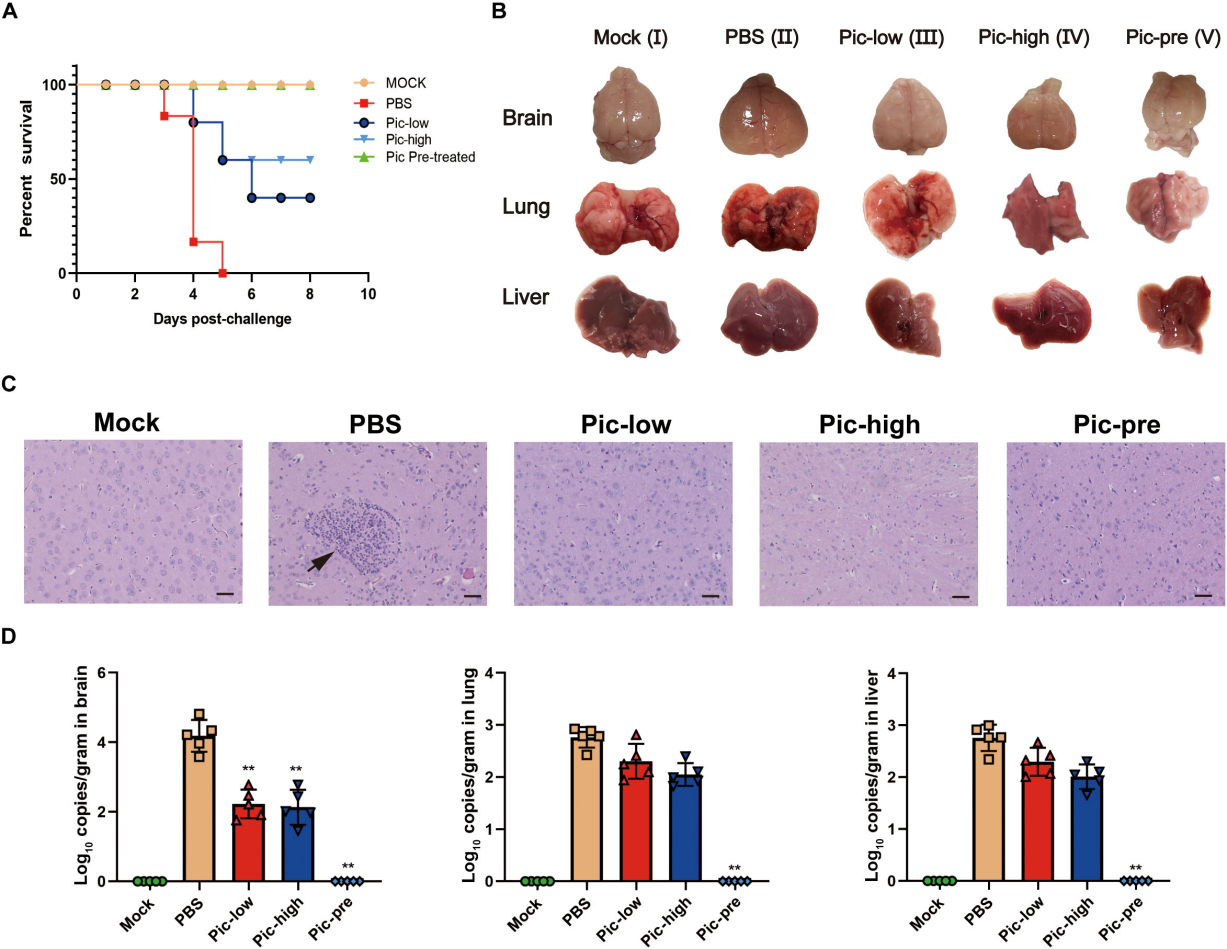
Protective effect of piceatannol against nasal PRV infection. (A to C) The protective effect of piceatannol against nasal PRV infection was evaluated across the following groups (*n* = 11 per group): blank control (Mock), PRV challenge (PBS), low-dose piceatannol nasal inoculation group (20 mg/kg) (Pic-low), high-dose piceatannol nasal inoculation group (50 mg/kg) (Pic-high), and piceatannol-pretreated virus nasal infection group (Pic-pre). (A) Survival curve of mice in each experimental group. (B) Gross pathological examination of brain, lung, and liver tissues from mice in each experimental group. (C) Histopathological sections of mouse brain tissues from each experimental group, with black arrows indicating inflammatory cell infiltration (scale bar, 50 μm). (D) Viral load in brain, lung, and liver tissues of mice in each experimental group, as detected by qPCR. All data are presented as means ± SD, with comparisons performed by one-way ANOVA. *P < 0.05, **P < 0.01.

The in vivo challenge protection experiment further validated the antiviral efficacy of piceatannol. Compared to the PRV challenge group, nasal piceatannol administration significantly decreased the mortality rate in mice infected with PRV, with survival rates of 50% and 66.7% observed in the low- and high-dose cohorts, respectively (Fig. 7A). Histopathological analysis revealed that piceatannol markedly mitigated hemorrhagic events in both cerebral and pulmonary tissues and reduced inflammatory cell infiltration within cerebral tissue induced by PRV infection (Fig. 7B and C). The RT-qPCR results indicated significantly lower viral loads in the brain, lungs, and liver of the piceatannol-treated group than in those of the PRV-challenged group (Fig. 7D). Notably, the piceatannol-pretreated group exhibited a 100% survival rate with no pathological changes in the brain and lung tissues, and no PRV was detected in the brain, lung, or liver of the piceatannol-pretreated group (Fig. 7A to D).

### Piceatannol prevents host cell invasion by disrupting viral membrane fusion

**Fig. 8.**
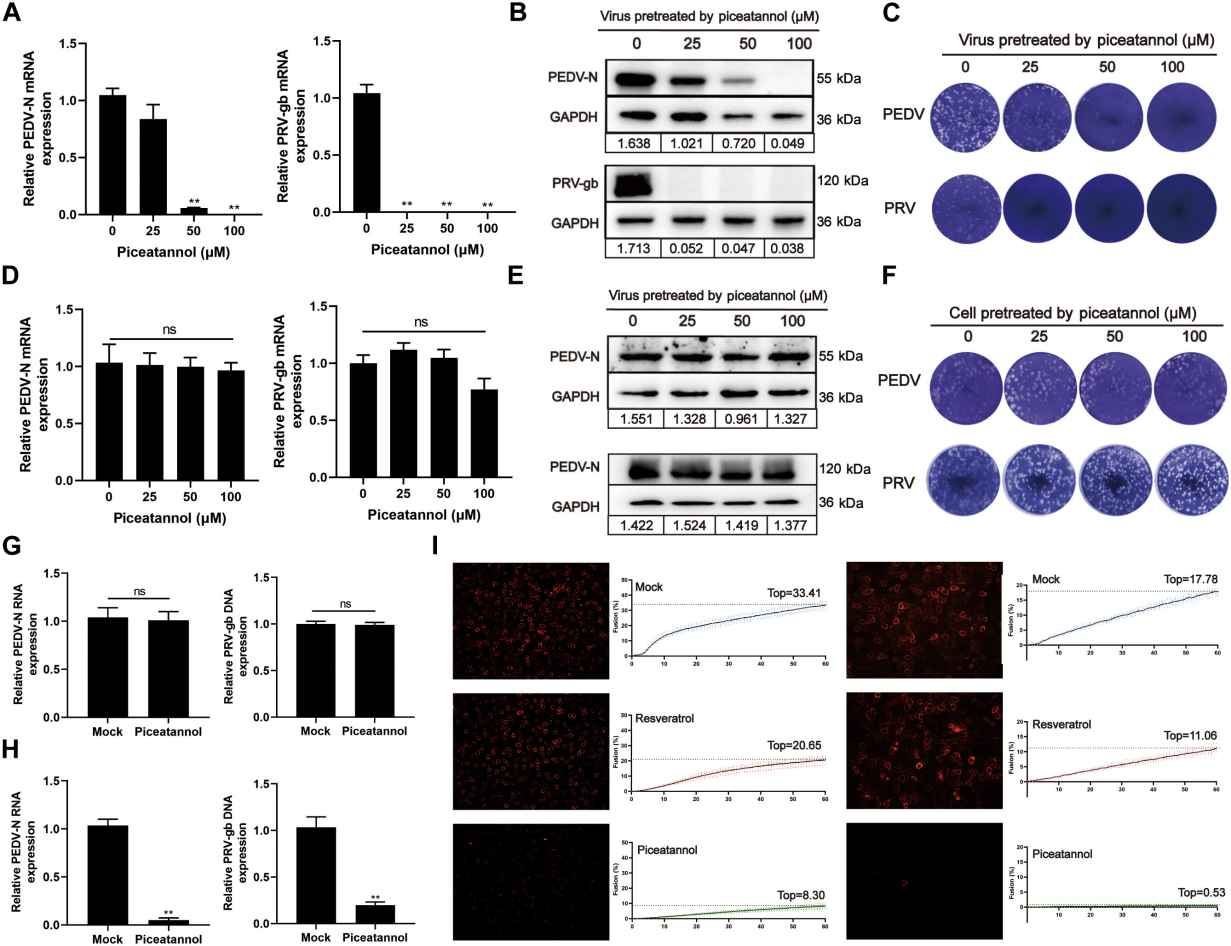
Piceatannol exerts direct antiviral activity by inhibiting viral membrane fusion. (A to C) To verify the direct antiviral effect of piceatannol on PEDV and PRV, PEDV or PRV (0.1 MOI) was incubated with piceatannol at 0, 25, 50, or 100 μM at 37 °C for 1 h, followed by infection of Vero E6 or PK15 cells. Viral gene and protein expression were analyzed using RT-qPCR (A) and Western blot (B) after 24 h, and plaque assays were used to evaluate the antiviral effects (C). (D to F) To evaluate whether piceatannol pretreatment of cells prevents viral infection, Vero E6 or PK-15 cells were treated with 0, 25, 50, or 100 μM piceatannol for 1 h. Piceatannol was then removed with blank DMEM, and the cells were inoculated with 0.1 MOI of PEDV or PRV followed by assessment of viral gene (D) and protein (E) expression using RT-qPCR and Western blot after 24 h. Supernatant viral titers (F) were determined using a plaque assay. (G) To assess the impact of piceatannol pretreatment on viral attachment, PEDV and PRV were incubated with piceatannol in Vero E6 or PK-15 cells at 4 °C for 30 min. Unattached viruses were removed, and viral RNA was detected using RT-qPCR. (H) To evaluate the impact of piceatannol on receptor-mediated cell entry, PEDV and PRV were treated with piceatannol and incubated with Vero E6 or PK-15 cells at 4 °C for 30 min. Unbound viruses were removed, and cells were incubated at 37 °C for 1 h and washed with pH 3 citrate buffer for viral RNA measurement using RT-qPCR. (I) Octadecyl rhodamine (R18) was used to assess the impact of piceatannol on viral membrane fusion. R18 is initially nonfluorescent but becomes fluorescent during lipid rearrangement under fusion, allowing real-time tracking. R18-labeled PEDV or PRV was first exposed to piceatannol, resveratrol, or a solvent control at 37 °C for 10 min and incubated with Vero E6 cells at 4 °C for 1 h while monitoring fluorescence signals. Triton X-100 was added to each well to a final concentration of 0.5%, and fluorescence was remeasured after incubation at 37 °C for 1 h to obtain the maximum fluorescence (*F*_max_). The fusion rate was calculated as Fusion rate (%) = (*F*_x_ − *F*_initial_)/(*F*_max_ − *F*_initial_), where *F_x_* denotes real-time fluorescence intensity, *F*_initial_ represents initial fluorescence intensity, and *F*_max_ is the maximum fluorescence intensity. The *x* axis represents time, and the *y* axis represents membrane fusion, with data presented as the mean ± SD from 7 experiments and fitted to a sigmoidal curve to calculate the maximum fusion. The right panel shows fluorescence microscopy images of treated groups without Triton X-100, with red fluorescence indicating membrane fusion. All data are presented as means ± SD, and comparisons were performed using one-way ANOVA. *P < 0.05, **P < 0.01.

Building on studies demonstrating the antiviral activity of piceatannol using plaque reduction assays, we aimed to identify the target stage for piceatannol in viral infections. Two experimental conditions were evaluated: pretreatment with the virus and pretreatment with host cells. Piceatannol was thus incubated with PEDV and PRV at 37 °C for 1 h, followed by a 100-fold dilution and inoculation with the treated viral suspensions onto susceptible cell cultures. As shown in Fig. 8A and B, pretreatment with piceatannol significantly reduced viral RNA transcription and protein expression in infected cells. Moreover, the levels of infectious viral particles in the culture supernatant were markedly decreased (Fig. 8C). However, piceatannol did not significantly affect PEDV replication or viral release, as indicated by the unchanged transcription levels of key viral genes, viral protein expression, and infectious particle production (Fig. 8D to F). Further analysis was aimed at identifying the precise stage at which piceatannol exerts its antiviral effects. As illustrated in Fig. 8G, pretreatment with piceatannol significantly inhibited the entry of PEDV and PRV into Vero E6 and PK-15 cells, whereas viral adhesion to host cells remained unaffected (Fig. 8H). Because PEDV and PRV primarily invade host cells through receptor-mediated membrane fusion, these results suggest that piceatannol disrupts the membrane fusion process required for viral entry. To assess the specificity of this inhibition, porcine circovirus type 2 (PCV2), a non-enveloped virus that enters cells via receptor-mediated endocytosis, was used as a control. Piceatannol treatment did not noticeably affect PCV2 infection, confirming the selectivity of its antiviral effects toward the membrane fusion entry pathway of enveloped viruses (Fig. S12).

The potential of piceatannol to enhance antiviral activity by modulating host cell mechanisms was also explored. To assess the inhibition of membrane fusion, an assay using octadecyl rhodamine, a lipophilic dye used to detect fusion through changes in fluorescence intensity, was conducted. As shown in Fig. 8I, gradually increased fusion was observed for untreated PEDV with Vero E6 cells, reaching 33.41%. In contrast, piceatannol pretreatment significantly reduced fusion to 8.30%, indicating strong inhibition, whereas resveratrol had a minimal effect. Similarly, PRV fusion with PK-15 cells reached 17.78% in the untreated group; however, piceatannol pretreatment nearly abolished the fusion completely, reducing it to 0.53%.

### Piceatannol inhibits viral membrane fusion by directly targeting viral envelope lipid molecules

**Fig. 9.**
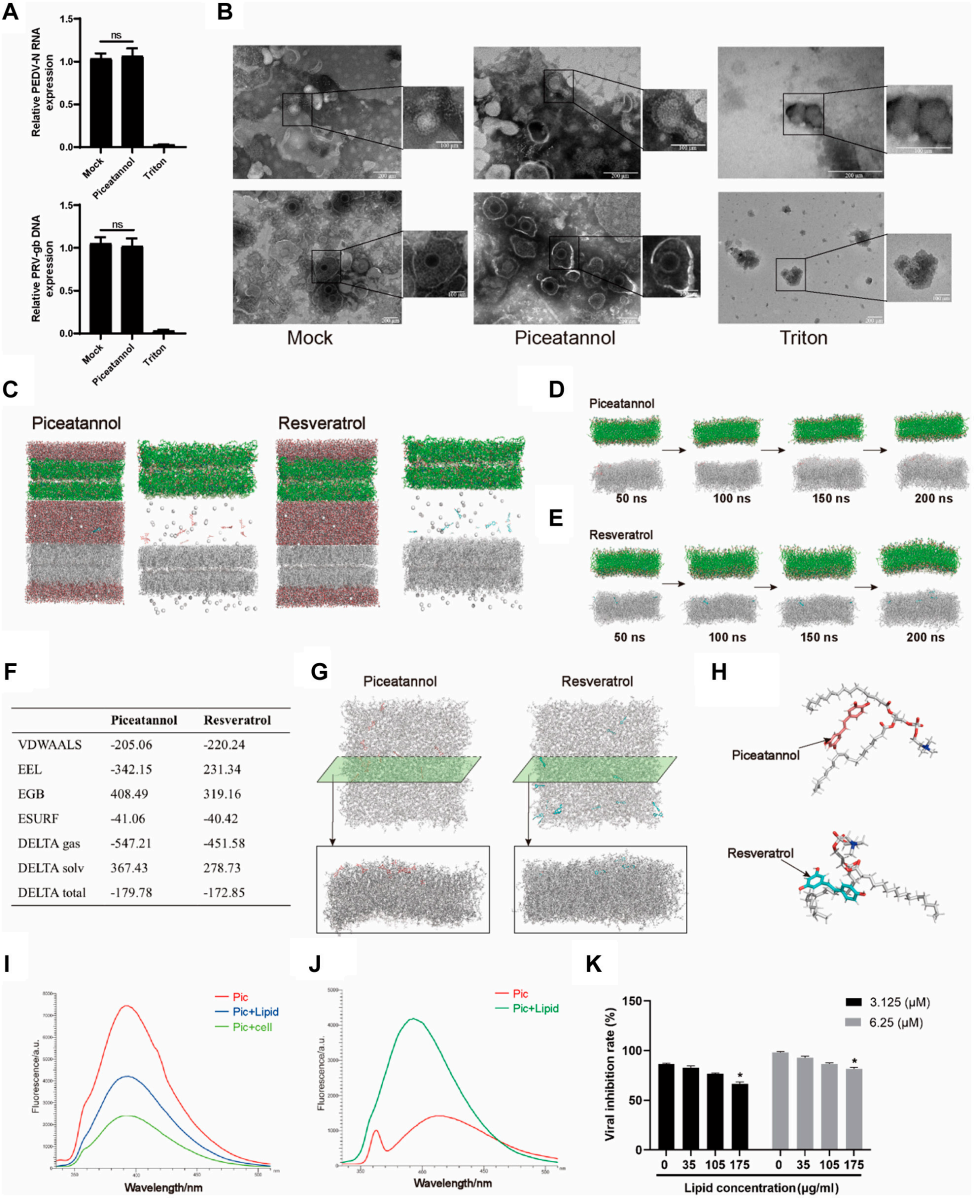
Piceatannol penetrates the viral envelope effectively without compromising its integrity. (A) Viral nucleic acid exposure assay in which PEDV or PRV was incubated with piceatannol for 1 h, followed by treatment with RNase A or DNase I to selectively degrade exposed nucleic acids. The total viral nucleic acids were then extracted and quantified using RT-qPCR. DMSO was used as the solvent control (negative), while Triton X-100 served as the positive control to induce envelope disruption. (B) Transmission electron microscopy analysis of PEDV and PRV particles treated with piceatannol, with DMSO and Triton X-100 as controls. Scale bar, 200 nm. Magnified images illustrate both intact and disrupted viral particles. (C to F) MD simulation analysis of the interaction between piceatannol and the viral envelope. The bilayer model was constructed using Packmol and consisted of an upper viral envelope and a lower cellular membrane. Piceatannol or resveratrol was randomly distributed between the bilayers (C), followed by 100-ns MD simulation under standard temperature and pressure conditions (D and E). Viral envelope lipids are depicted as gray sticks, phospholipids as green sticks, water molecules as red lines, and counter ions as white spheres. Piceatannol is represented by blue sticks, and resveratrol as red sticks. (F) Binding free energy calculated from MD simulations. VDWAALS, van der Waals energy; EEL, electrostatic energy; EGB, polar solvation energy; ESURF, nonpolar solvation energy; Δ*G*_gas_, total gas-phase free energy; Δ*G*_solv_, total solvation free energy; Δ*G*_total_, total binding free energy. The formula Δ*G*_total_ = Δ*G*_solv_ + Δ*G*_ga_ was used. (G) Detailed view of piceatannol penetration into the viral envelope, with both top-down (planar) and side (cross-sectional) views. (H) Interaction details of piceatannol and PC within the viral envelope, highlighting the molecular interactions after penetration. (I) Piceatannol was incubated with cell-derived lipids and centrifuged to remove unbound piceatannol. Vero E6 cells served as a control. Methanol was used to elute the bound piceatannol, and fluorescence was measured at 320 nm. The binding rates to cells and lipids were 32.4% and 56.5%, respectively. (J) Piceatannol was incubated with cell lipids, causing the fluorescence peak to shift from 410 nm in PBS to 391 nm, with increased intensity indicating binding of piceatannol to lipid molecules. (K) Piceatannol was incubated with varying concentrations of lipids, followed by a plaque inhibition assay to assess antiviral activity against PRV. All data are presented as means ± SD, and comparisons were performed using one-way ANOVA. *P < 0.05, **P < 0.01.

Following the observation that piceatannol inhibits membrane fusion in enveloped viruses, the possibility that piceatannol compromises the viral envelope was investigated via a nucleic acid exposure assay. Triton X-100, serving as a positive control, successfully disrupted the viral envelope, resulting in markedly decreased nucleic acid levels compared to the solvent control, as confirmed by fluorescence quantification. In contrast, piceatannol treatment did not appreciably alter nucleic acid levels relative to the solvent control, indicating that piceatannol did not induce viral envelope disruption (Fig. 9A). To assess the structural integrity of the viral envelope, transmission electron microscopy was used to investigate PEDV and PRV particles. The viral envelope was visibly disrupted and intact viral particles were absent in the Triton X-100-treated group; however, piceatannol-treated samples displayed viral envelopes that were indistinguishable from those of the solvent control, indicating no detectable structural damage (Fig. 9B). Consistent with the results of the nucleic acid exposure assay, these observations suggest that the antiviral mechanism of piceatannol does not involve direct envelope disruption.

To elucidate the effects of piceatannol on viral and host membrane interactions, molecular dynamics (MD) simulations were performed using resveratrol, a structurally similar compound without antiviral activity, as a control. A double-bilayer model representing the viral envelope and host cell membrane was constructed with phosphatidylcholine (PC), phosphatidylethanolamine (PE), and phosphatidylinositol (PI) at a 6:2:2 ratio. Ten molecules of piceatannol or resveratrol were randomly distributed between the viral envelope and host cell membrane to evaluate their influence on the membrane dynamics and interactions, as seen in Fig. 9C, with both compounds being gradually integrated into the membrane bilayers (Fig. 9D and E). Additional structural dynamic metrics derived from the MD simulations indicated that piceatannol exerts a more pronounced effect on membrane structure than resveratrol. Root mean square deviation analysis revealed greater fluctuations and decreased stability in the piceatannol system (Fig. S13A), whereas the radius of gyration (Rg) indicated stronger membrane perturbations in the presence of piceatannol (Fig. S13B). Although the root mean square fluctuation (RMSF) values did not change significantly, the overall results suggest that piceatannol substantially increases both membrane instability and structural dispersion (Fig. S13C).

Binding free energy calculations under dynamic simulation conditions showed that both piceatannol and resveratrol possess negative binding free energies, reflecting spontaneous energetically favorable interactions with the viral envelope. Notably, piceatannol displayed a lower binding free energy (−179.78 kcal/mol) than resveratrol (−172.85 kcal/mol), indicating stronger binding affinity. Free energy decomposition revealed that electrostatic interactions were the primary contributors to this difference, likely due to the additional hydroxyl group in piceatannol, which enhances polar interactions and substantially increases its membrane-binding affinity (Fig. 9F).

The 2-dimensional planar projections in the final state indicated that piceatannol achieved greater insertion depth and abundance within the phospholipid bilayer of the viral envelope than resveratrol (Fig. 9G), suggesting stronger membrane affinity and more effective inhibition of membrane fusion. Furthermore, as shown in Fig. 9H, piceatannol molecules tended to cluster near the double bonds in the phospholipid alkyl chains following integration into the viral envelope, implying specific interactions with viral lipids. Collectively, these findings indicate higher affinity and deeper membrane insertion for piceatannol than for resveratrol. Given that the lipid components of the viral envelope originate in the host cell membrane, membrane lipids from Vero E6 cells were isolated to investigate the interactions between piceatannol and lipids. Specifically, piceatannol was incubated with either intact Vero E6 cells or isolated lipids at 37 °C for 1 h and then centrifuged to remove unbound components. The bound fraction was then eluted with methanol, and the amount of bound piceatannol was quantified by fluorescence detection at an excitation wavelength of 320 nm. The results showed that piceatannol had a higher binding affinity for lipids (56.5%) than for intact cells (32.4%) (Fig. 9I). Subsequent fluorescence analysis revealed a blue shift in the emission peak from 410 nm in phosphate-buffered saline (PBS) to 391 nm following incubation with lipids, indicating strong interaction between piceatannol and the lipid components (Fig. 9J). Plaque inhibition assays were then used to evaluate the influence of lipid binding on the antiviral efficacy of piceatannol, revealing that 3.125 μM piceatannol inhibited PRV by 86.3%. However, the addition of lipids at a concentration of 175 μg/ml markedly reduced this inhibition to 68.9% (Fig. 9K). These findings indicate that although piceatannol exhibits a strong binding affinity for viral envelope lipids, this interaction ultimately diminishes its antiviral activity.

## Discussion

Viral respiratory infections pose a serious threat to both animal and human health, presenting persistent challenges to agricultural and public health systems. Recent studies have indicated that enhanced nasal mucosal immune protection can effectively improve disease resistance against severe acute respiratory syndrome coronavirus 2 (SARS-CoV-2), influenza, and other respiratory pathogens [28]. This study underscores the beneficial effects of outdoor exposure on the respiratory health of pigs, as evidenced by epidemiological monitoring. Compared with fully confined pigs, those with outdoor access exhibited markedly enhanced immune protection in the nasal mucosa, with notably reduced pathogen detection. Further investigation revealed that this protective effect is closely associated with the colonization of *B. subtilis* in the nasal mucosa, which secretes antiviral metabolites that directly obstruct the viral invasion of host cells.

Although conventional wisdom often attributes the increased incidence of infectious diseases in intensive farming to overcrowding and inadequate hygiene, addressing these factors alone is insufficient for effectively controlling pathogen transmission [1]. Previous studies have shown that intensive farming practices substantially influence the mucosal microbiota of animals, compromising their immunity and enhancing their susceptibility to pathogens [29]. In this study, exposure to outdoor environments was associated with a marked increase in the overall diversity of porcine nasal microbiota; in particular, the relative abundances of taxa from the phyla Firmicutes and Actinobacteria were notably increased, and certain potentially pathogenic members of the phylum Proteobacteria were decreased [30–32]. These findings suggest that outdoor activities optimize the nasal microbial community, promoting beneficial bacteria and inhibiting pathogen proliferation, thus improving respiratory health. Although lactic acid bacteria, a subgroup of Firmicutes known to support mucosal barriers and enhance immunity, was also increased in pigs with outdoor access, our results indicated that only colonization with *Bacillus* species was negatively correlated with pathogen detection rates. A Gram-positive bacterium that is commonly found in soil, water, and plant surfaces, *Bacillus* forms heat-resistant spores, enabling it to adapt to diverse environmental conditions and become a prevalent member of microbial communities [33]. Outdoor-accessing pigs interact with the soil, water, and vegetation during rooting and foraging, and likely introduce environmental *Bacillus* spp. into the nasal mucosa via these activities. This suggests that outdoor activities not only modify porcine behavior but also reshape microbial communities through interactions between environmental microbes and the host.

The probiotic properties of *Bacillus* spp. have attracted considerable attention in recent years, with research demonstrating that *Bacillus* (primarily *B. subtilis*) can effectively reduce methicillin-resistant *S. aureus* colonization in rural populations in Thailand, primarily through the secretion of antimicrobial peptides known as fengycins [34]. *B. subtilis*, recognized as the most extensively researched probiotic species within its genus, facilitates nutrient absorption, mitigates intestinal inflammation, and modulates gut immune function [35,36]. Recent work reveals that its metabolite 2-hydroxy-4-methyl-pentanoic acid reinforces the intestinal epithelial barrier by triggering the GADD45A-dependent Wnt/β-catenin signaling cascade [37]. However, as an aerobic bacterium, *B. subtilis* enters a dormant spore state when oxygen is depleted, leading to the loss of its long-term probiotic effects [38]. The oxygen-rich environment and absence of digestive enzymes on the nasal mucosal surface thus provide an ideal habitat for *B. subtilis*, allowing it to maintain its probiotic activity. Our previous study demonstrated that the administration of *B. subtilis* 168 increased the number of immune cells and the expression of pattern recognition receptors in nasal lymphoid tissue, enhancing the innate immune response of the nasal mucosa [39]. In this study, *B. subtilis* with broad-spectrum antiviral activity was isolated from the nasal mucosa of pigs with outdoor access, further highlighting its multifunctional protective role. Of the isolates obtained, NS12 not only exhibited the strongest broad-spectrum antiviral activity, but also showed pronounced mucosal colonization, which is crucial for its sustained protective effect in the nasal mucosa. As an environmental isolate, the ability of *B. subtilis* NS12 to colonize the nasal mucosal surface may be enhanced by the formation of biofilms, metabolic adaption to the local environment, and modification of its cell wall and outer membrane proteins to evade immune recognition [40]. Some studies have shown that respiratory probiotics, such as *Lactobacillus rhamnosus*, *Bifidobacterium*, and *Lactobacillus salivarius*, substantially reduce the severity and duration of infections caused by influenza, SARS-CoV-2, RSV, and Norwalk virus. These probiotics protect the respiratory system by enhancing mucosal immunity, including strengthening epithelial junctions, activating immune cells, increasing antiviral cytokines [e.g., interferon-γ (IFN-γ) and interleukin-12 (IL-12)], and promoting neutralizing antibody production [40–43]. However, these protective mechanisms are typically triggered after viral infection, limiting their roles in early defense. In contrast, the *B. subtilis* NS12 isolated in this study secretes metabolites with broad-spectrum antiviral activity, forming an effective defense barrier on the mucosal surface that directly blocks viral entry. This unique capability enhances the overall resistance of the respiratory mucosa and effectively inhibits the transmission and spread of viruses following infection.

Of the diverse natural products synthesized by *B. subtilis*, lipopeptides such as surfactin, Zycin, and iturin are known to have substantial antibacterial, antiviral, and immunomodulatory activities [44]. This study demonstrated remarkable stability for the *B. subtilis* NS12 supernatant, with robust antiviral activity at low temperatures (4 °C), which is consistent with the known properties of surfactin. Surfactin, a cyclic lipopeptide comprising 7 amino acid residues and a fatty acid side chain, possesses a unique structure with substantial chemical and physical stability, enabling it to retain considerable activity even under adverse conditions [45]. Although the antiviral activity of surfactin was first identified in the 1990s, the molecular mechanisms underlying this activity were preliminarily elucidated by our research team in 2018 [26]. Specifically, we found that the inverted cone-shaped molecular structure of surfactin interfered with the fusion of viruses with host cell membranes. Compared with standard surfactin, the surfactin that was extracted from NS12 demonstrates broad-spectrum antiviral activity with marked inhibitory effects against PRV and exhibits a wider safe concentration range, suggesting its enhanced potential for therapeutic applications. This is in contrast to standard surfactin, which induces hemolysis and cytotoxicity at effective concentrations. The structural characteristics of surfactin, particularly the length of the fatty acid chains and the arrangement of amino acids within the peptide sequence, are essential for its antiviral activity [46]. MS analysis indicated that the surfactin produced by NS12 is compositionally comparable to the standard but contains 2 novel C16 and C17 surfactin derivatives (*m*/*z* = 1,054 and 1,068). We hypothesize that these novel homologs may enhance the safety profile and favorable antiviral activity of the NS12 strain; however, further experimental validation is warranted. Under typical conditions, *B. subtilis* allocates its energy primarily to maintaining its basic metabolic activities and reproduction, leading to low lipopeptide compound yields [47]. However, the NS12 strain isolated in this study demonstrated a strong capacity to synthesize surfactin in standard LB medium, with production levels similar to those of the recombinant strain *B. subtilis* OKB105, which achieved high surfactin synthesis via the introduction of the *sfp* gene into *B. subtilis* 168 [48]. We hypothesize that the growth of the NS12 strain within the nasal mucosa may confer unique metabolic characteristics due to genetic variations in the lipopeptide synthesis genes, enabling it to sustain high surfactin production even under nonstress conditions. Optimization of the fermentation conditions for NS12 has the potential to greatly enhance surfactin production, facilitating the large-scale and efficient production of such natural antiviral products.

*B. subtilis* not only produces lipopeptides but also generates a variety of biologically active secondary metabolites such as ketones, alcohols, acids, and aromatic compounds [49]. Nontargeted metabolomic analysis revealed the presence of markedly higher levels of several antiviral small molecules, such as daidzein, α-tocopherol, benzoic acid, biochanin A, resveratrol, and piceatannol, in the culture supernatant of the NS12 strain as compared to the non-antiviral *B. subtilis* 168 strain. Notably, piceatannol exhibited strong direct antiviral activity at effective concentrations below 10 μM. As a phenolic compound, piceatannol is known for its anti-inflammatory, antioxidant, and antitumor properties and has recently been reported to exhibit antiviral activity against cytomegalovirus, although the underlying mechanisms remain unclear [50,51]. Our study demonstrated that piceatannol can effectively block fusion between the viral envelope and the host cell membrane without disrupting the structure of the viral envelope, inhibiting viral entry into host cells. Interestingly, although resveratrol differs from piceatannol by only one hydroxyl group and has been reported to exert broad-spectrum antiviral effects by modulating multiple cellular signaling pathways [52], the efficacy of resveratrol in blocking viral entry or inhibiting membrane fusion was not observed in this study. This indicates that even minimal structural differences can lead to pronounced variations in the antiviral mechanisms of a compound. Therefore, future studies should focus on elucidating the relationship between structural differences and functional outcomes to further optimize the antiviral properties of these compounds and expand their potential applications in antiviral drug development.

Previous studies have shown that piceatannol and resveratrol can be integrated into PC-based lipid membrane models, modulating the membrane fluidity and phase transition temperature. Notably, piceatannol exhibited a more pronounced effect on the phase transition temperature than resveratrol [53]. Considering that the viral envelope originates in the host cells and consists of a bilayer structure composed of PC, PE, and PI, with different hydrophilic heads and hydrophobic tails [54], we hypothesize that resveratrol interacts with the viral phospholipid bilayer in a similar manner. MD simulation indicated that piceatannol penetrates the lipid bilayer of the viral envelope more effectively than resveratrol, primarily binding near the double bonds in fatty acid chains. Fluorescence spectroscopy further confirmed the affinity of resveratrol for phospholipids, and phospholipid pretreatment significantly reduced the antiviral efficacy of piceatannol. Membrane fusion occurs when 2 separate lipid bilayers merge into a continuous bilayer and involves hemifusion intermediates, fusion pore expansion, and complete membrane integration. This process is influenced by the shape and structure of the phospholipid molecules [55], and negatively charged, conical, highly fluid, and high-curvature phospholipids have been confirmed to promote fusion [56]. Although MD simulations have shown that most piceatannol molecules that penetrate the viral envelope are localized near the double bonds of the PC fatty acid chains, some studies have indicated that piceatannol tends to interact with the headgroup regions of phospholipids [53]. Therefore, quantum chemical calculations were used to separately investigate the reactivity of piceatannol with the double bonds and headgroup regions of phospholipid molecules. The results indicates that piceatannol is far more reactive than resveratrol: The radical oxidation barrier is −23.8 kcal/mol versus 0.3 kcal/mol. This heightened reactivity arises from piceatannol’s C-4 ortho-dihydroxy motif, which increases electron delocalization and polarity. As a result, piceatannol adds to the double bonds of viral phospholipid acyl chains with a lower energetic cost than resveratrol (48.1 versus 50.9 kcal/mol for PC), raising lipid saturation and bilayer rigidity (Fig. S14A). Given that unsaturated fatty acids are crucial for maintaining fluidity in model membranes, the incorporation of resveratrol may increase the rigidity of the viral envelope phospholipid bilayer, reducing membrane fluidity and inhibiting membrane fusion [57,58]. Additionally, computational results revealed that resveratrol is more inclined to engage in choline transfer reactions with phospholipid headgroups, increasing the headgroup volume of the phospholipids (such as PI and PE) and causing a shift in their conformation from cylindrical or conical to cone-like structures similar to lysolipid PC and lysolipid phosphatidylglycerol (Fig. S14B to D), introducing a positive curvature and further impeding membrane fusion [55].

In summary, *B. subtilis* NS12, demonstrating broad-spectrum and highly efficient antiviral activity, was isolated from the nasal mucosa of outdoor-accessing pigs and observed to exhibit robust respiratory mucosal resistance to pathogens. The molecular mechanisms through which NS12 secretes novel lipopeptides and the phenolic compound piceatannol, thereby inhibiting the viral invasion of host cells, were thoroughly elucidated. Future research will delineate the precise molecular interactions between the *B. subtilis* NS12 metabolites and viral components and explore their synergistic effects with existing antiviral therapies. This probiotic approach is a promising candidate for innovative antiviral strategies with considerable potential to mitigate viral respiratory infections in agriculture and public health sectors.

## Conclusion

**Fig. 10.**
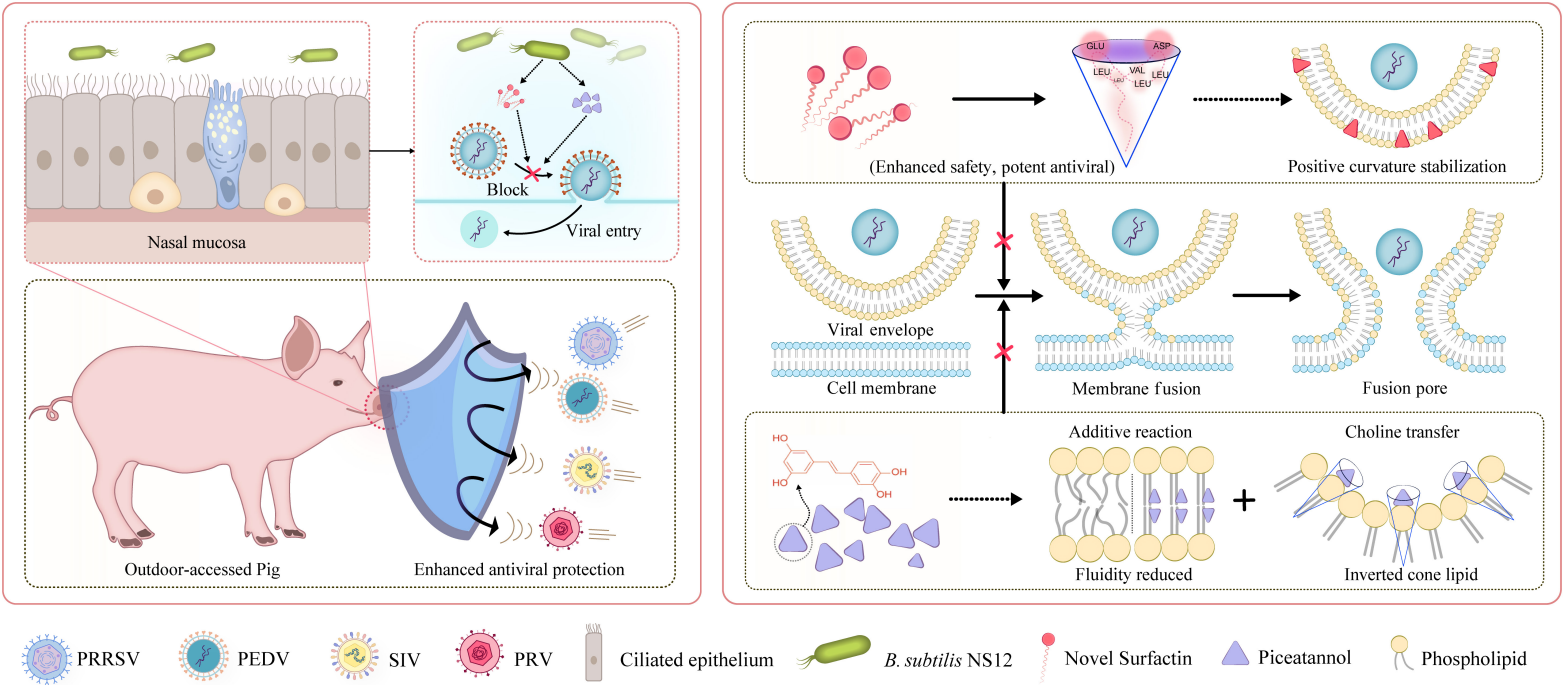
Protective mechanism of broad-spectrum antiviral defense conferred by nasal mucosa-colonized *B. subtilis* NS12.

Respiratory infectious diseases pose serious challenges to both livestock and public health. Our findings highlight the enhanced resistance of outdoor-accessing pigs to respiratory infections and demonstrate that nasal colonization with *Bacillus* species plays a crucial role in mucosal protection. *B. subtilis* NS12, isolated from the nasal cavities of outdoor-accessing pigs, effectively colonizes mucosal surfaces and exhibits strong antiviral properties. The synthesis of distinct antiviral compounds, including a novel variant of surfactin and the phenolic compound piceatannol, was characterized by improved safety and antiviral efficacy. These compounds inhibit viral entry into host cells by modulating the saturation, rigidity, fluidity, and headgroup volume of phospholipid molecules within the viral envelope (Fig. 10). Consequently, this mechanism impedes the fusion of viral envelopes with cellular membranes. Thus, this probiotic is a promising candidate for developing innovative and environmentally sustainable antiviral agents for the prevention and management of respiratory infections in animals and humans, negating reliance on vaccines. Moreover, because the lipid bilayer of the viral envelope originates from host cells, the potential for developing resistance due to viral mutations is minimal. Metabolites produced by NS12, which interact with viral membrane phospholipids and disrupt membrane fusion, hold substantial promise as potent inhibitors of enveloped viral infection.

## Materials and Methods

### Reagents

Piceatannol, resveratrol, daidzein, α-tocopherol, atropine, chickpea isoflavone A, and p-hydroxycinnamic acid were purchased from TCI, Japan; benzoic acid was obtained from Macklin Biochemical Technology (Shanghai, China). Surfactin standards for high-performance liquid chromatography (HPLC) analysis were obtained from Sigma-Aldrich Labor Chemikalien GmbH (Seelze, Germany). PCV2 and capsid antibody (catalog no. GTX128121) were purchased from GeneTex International Corporation. The anti-PRRSV N protein monoclonal antibody was provided by P. Li of Yichun University. The anti-PEDV N protein monoclonal antibody was custom-made by Jinsirui Biotechnology Co. Ltd. (Nanjing, China) and was preserved in our laboratory (diluted to 1:200 for IF and 1:1,000 for Western blot analysis). The anti-PRV gB-protein monoclonal antibody was provided by P. Jiang at Nanjing Agricultural University. Secondary antibodies for IF, including goat anti-rabbit Alexa Fluor 488 and goat anti-mouse Alexa Fluor 594, were purchased from Invitrogen (Carlsbad, CA, USA). Diamidino-2-phenylindole (DAPI; 1:1,000, 2313070) was purchased from Thermo Fisher Scientific (Waltham, MA, USA). All other reagents and chemicals were purchased from Sigma-Aldrich (St. Louis, MO, USA).

### Viruses, cell lines, and bacterial strains

The PRRSV strain JXwn06 (GenBank: EF641008.1) was provided by P. Li at YiChun University. The PRV strain ZJ01 (GenBank: KM061380.1) and PCV2 strain SH (GenBank: AY686763.1) were provided by P. Jiang at the College of Veterinary Medicine, Nanjing Agricultural University. The influenza virus *H1N1* strain A/swine/Guangdong/1/2011 (GenBank: MT410579) was kindly provided by Z. Feng from the Jiangsu Academy of Agricultural Sciences. The wild-type PEDV strain Zhejiang08, which clusters with the emerging virulent strain (GenBank: JX002693), is preserved in our laboratory. Recombinant PEDV-expressing GFP was provided by Z. Li at the Institute of Veterinary Medicine, Shanghai Academy of Agricultural Sciences [59]. Vero E6 cells were used for the propagation and titration of PEDV, PK15 cells for the propagation and titration of PRV, and MARC-145 cells for the propagation and titration of PRRSV, while A549 cells were used for propagating the influenza virus and Madin-Darby canine kidney (MDCK) cells for virus titration. All cell lines were preserved in our laboratory and were regularly tested for mycoplasma contamination. They were cultured in Dulbecco’s modified Eagle’s medium (DMEM) supplemented with 10% fetal bovine serum (FBS) and incubated at 37 °C in a humidified atmosphere containing 5% CO₂. For viral inoculation, a confluent monolayer of target cells was inoculated with different viruses at a multiplicity of infection (MOI) of 1 for 1 h at 37 °C. The inoculum and unattached viruses were removed by washing with DMEM. Maintenance medium (DMEM with 2% FBS) was then added, and the culture was incubated at 37 °C under 5% CO₂. Infected cells were analyzed after the required incubation period. To harvest viruses, infected cells were subjected to one freeze–thaw cycle, achieving a cytopathic effect of 80%, and the cell supernatant was collected. All viral particles were purified using sucrose density gradient centrifugation and titrated onto Vero cells using a plaque assay. Following each purification, the viral protein concentrations were determined using bicinchoninic acid (BCA) assay protein reagents, and the purified virus was preserved at −80 °C. *B. subtilis* NS12 and the reference strain *B. subtilis* 168 (American Type Culture Collection, 23857) were cultured in LB broth (10 g/l tryptone, 5 g/l yeast extract, 10 g/l NaCl; pH 7.0) or on LB agar plates (LB broth supplemented with 1.5% agar). A single colony was inoculated into LB broth and grown overnight at 37 °C, 220 rpm, then subcultured (1% v/v inoculum) into fresh LB broth, and incubated at 37 °C, 220 rpm for 14 h (triplicate flasks containing 100 ml of medium in 250-ml baffled flasks). Cells were harvested at OD₆₀₀ (optical density at 600 nm) ≈ 0.8 to 1.0 by centrifugation (8,000*g*, 10 min, 4 °C), and supernatants were filter-sterilized using 0.22-μm polyethersulfone (PES) filters. Bacterial cells were subsequently inactivated by high-pressure steam sterilization (121 °C, 20 psi, 20 min).

### Animals

Pigs from 2 medium-sized farms in the hilly regions of eastern China were monitored for respiratory viruses. Over the past 3 years, both farms have documented the occurrence of respiratory and gastrointestinal infectious diseases associated with various pathogens, including SIV, PRRSV, PRV, and PEDV. Traditionally, both farms use closed, intensive farming models. However, recent modifications have introduced free-range areas that spans approximately 25 acres of enclosed hillsides in which weaning and fattening pigs can roam freely among the trees, with a spacious open-sided shelter available for resting. From each farm, 60 clinically healthy 30-day-old nursery pigs were ear-tagged and block-randomized (1:1) to indoor- or outdoor-access groups by an independent biostatistician who used a published random-number table (block size = 6; entry point: row 18, column 11; read left to right). The allocation list remained sealed until tagging was complete to preserve concealment, and all personnel responsible for sampling and laboratory analyses were blinded to group assignments. The indoor-raised group was confined to traditional enclosures, while the outdoor-accessing group was subjected to a “confined with 6 h of daytime outdoor access” regimen, with 3 h of outdoor access in the morning and an additional 3 h in the afternoon. Both groups were provided with a standardized fattening diet that included unrestricted access to feed and clean water. Outdoor-accessing pigs received supplementary deworming treatment. The trial was conducted over a 1-year period during the spring and early summer, when average indoor temperatures ranged from 19 to 22 °C and outdoor temperatures from 16 to 25 °C.

One-day-old neonatal piglets were used for the PEDV challenge, while 4-week-old piglets were selected for the PRRSV challenge. The piglets in this study were sourced from a swine herd maintained by Peiqi Agricultural and Animal Husbandry Technology Co. Ltd. (Jiangsu, China), which is composed of cesarean-derived, colostrum-deprived piglets that test seronegative for antibodies against PEDV, PRRSV, porcine respiratory coronavirus, and transmissible gastroenteritis virus. To eliminate the potential influence of maternally derived antibodies, neonatal piglets were fed artificially throughout the experiment. Specific pathogen-free, 7-week-old female BALB/c mice obtained from the Animal Research Centre of Yangzhou University were used in the PRV challenge tests. All animals used in the study were of comparable weight and housed individually in separate rooms for 24 h prior to the study to allow for acclimatization to husbandry conditions and minimize stress. All animal experiments were approved by the Institutional Animal Care and Use Committee of Nanjing Agricultural University Animal Experiment Ethics Committee (NJAULLSC2021060 and NJAULLSC2022048, Nanjing, China).

### Analysis of viral presence and microflora composition in the nasal cavity of pigs

Various objective factors, such as accidental injuries and unexpected mortality, required the exclusion of monitored animals from each group during the study. Consequently, to maintain consistency in the sample size for the free-range and confined groups, nasal swabs were collected from 222 pigs (111 from each group) for pathogen detection, facilitating the comparison of nasal cavity infections in piglets subjected to both farming models. Nasal swabs were collected using a standardized procedure in which a sterile swab was inserted into the nasal vestibule and rotated 4 times with moderate pressure in each nostril. One swab sample was collected from both nostrils of each pig. Left nostril swabs were placed in viral transport medium and temporarily stored at −70 °C before elution in 1 ml of DMEM for viral detection, while right nostril swabs were immersed in 500 ml of 0.85% saline solution and temporarily stored at −20 °C for subsequent 16*S* rRNA sequencing and the detection, isolation, and identification of bacteria. RNA and DNA were extracted using a TIANamp Virus DNA/RNA Extraction Kit (Tiangen, China) and tested for SIV, PRRSV, PRV, and PEDV using RT-PCR using previously established protocols. The relevant primer sequences are listed in Table S1. Nasal swab samples were collected monthly throughout the trial, and detection results were categorized as follows: 0 for swabs with no pathogens recorded throughout the study period, 1 for the detection of a single pathogen, 2 for the detection of 2 pathogens, and 3 for the detection of 3 pathogens. The detailed detection results of viruses and *B. subtilis* in the nasal swab samples are listed in Data S1.

To examine the differences in the nasal mucosal microbiota of piglets raised under the 2 farming models, nasal swab samples from 25 indoor-raised piglets carrying multiple pathogens (i.e., with >1 pathogen detected) were pooled into 5 groups (*n* = 25), and samples from 25 randomly selected pathogen-free outdoor-accessing piglets were pooled into 5 groups for microbiota analysis. The composition of the nasal microbiota in the 10 pooled samples was subsequently assessed via 16*S* rRNA sequencing. Microbial DNA was extracted using a QIAamp DNA Microbiome Kit (Qiagen, Hilden, Germany) according to the manufacturer’s instructions. The V3–V4 hypervariable regions of the 16*S* rRNA gene were amplified using the primers 515F (5′-GTGCCAGCMGCCGCGGTAA-3′) and 806R (5′-GGACTACHVGGGTWTCTAAT-3′) following a standardized protocol for 16*S* amplicon library preparation. The PCR products were then verified using agarose gel electrophoresis, purified using the AMPure XP system (Beckman Coulter, Indianapolis, IN, USA), and quantified using a Qubit 3.0 Fluorometer (Thermo Fisher Scientific, Waltham, MA, USA). Purified amplicons were then pooled at equimolar concentrations and prepared for sequencing on an Illumina MiSeq platform (Illumina, San Diego, CA, USA), generating 250–base pair paired end reads. Raw sequences were filtered for errors and low-quality reads using the QIIME software, and clustering was performed using Vsearch (version 1.9.6), with sequences with 97% similarity grouped into operational taxonomic units (OTUs) for comparison with reference sequences in the Greengenes database and taxonomic assignment. Microbial community richness and diversity were evaluated using Chao1, SOB, and the Shannon index. Community analysis at the genus level was performed using Venn diagrams, while PCA was used to examine differences in the species composition across the samples. The taxonomic composition of each sample was analyzed across various levels, and a one-way analysis of variance (ANOVA) was performed on the experimental data using GraphPad Prism software.

### Detection, isolation, and colonization potency of *B. subtilis*

A total of 97 nasal swabs were collected from pathogen-free pigs with access to the outdoors. Eluate (100 μl) was extracted from each swab and heat-inactivated by incubation at 80 °C for 30 min to minimize contamination with non-spore-forming bacteria. The resulting eluates were then added to 5 ml of LB medium and incubated overnight at 37 °C while shaking at 220 rpm. *B. subtilis* was identified via the *gyrA* gene, which is specific to closely related species within the *B. subtilis* group. Positive bacterial cultures were then diluted and plated on LB agar, and single colonies exhibiting characteristic *B. subtilis* morphology were selected for isolation. Strain identification was confirmed using morphological analysis, biochemical characterization, and 16*S* rDNA sequencing. *B. subtilis* 168 was used as a positive control throughout the experiment. To evaluate the ability of *B. subtilis* to colonize mucosal surfaces, in vitro cell adhesion assays and in vivo nasal spray experiments were conducted. For the cell adhesion assay, carboxyfluorescein succinimidyl ester fluorescent dye-labeled *B. subtilis* was inoculated onto the surface of primary porcine nasal epithelial cells and cocultivated for 6 h before washing away nonadherent bacteria with DMEM. Fluorescence intensity was then quantified using a fluorometer to determine bacterial adhesion. In the nasal spray study, a spray containing 10^8^ CFU/ml of *B. subtilis* was administered to neonatal 5-day-old piglets that tested negative for nasal *Bacillus* spores. Nasal swabs were collected at days 3, 5, 7, 9, 11, and 14 post-inoculation. Because the administered *B. subtilis* strain lacked antibiotic resistance markers or fluorescent labels and no selective medium was available, we employed a heat selection enrichment method based on its heat-resistant spores [60]. Briefly, swab eluates were pasteurized (80 °C, 20 min) to eliminate vegetative bacteria, incubated in LB broth (37 °C, 2 h, shaking) to induce spore germination, and then enumerated by plate counting. Absolute quantitative PCR targeting the gyrA gene, specific for *Bacillus*, was performed in parallel as verification, using a standard curve of *y* = −0.3016*x* + 11.65 (*R*^2^ = 0.9939).

### Preliminary screening and evaluation of the antiviral activity of *B. subtilis*

A particular strain of the PEDV that expresses GFP was used for preliminary bulk screening to detect *B. subtilis* strains with antiviral activity. To focus on the prophylactic effects of *B. subtilis* against viral infections, 2 experimental setups were utilized based on previously established protocols: a preincubation virus group and a pretreatment cell group. In the preincubation experiment, isolated *B. subtilis* strains (10^5^ CFU) were co-incubated with PEDV [10^5^ plaque-forming units (PFU)] in 2% DMEM at 37 °C for 2 h. Following co-incubation, the culture supernatant was filtered using a 0.22-μm syringe filter (Millipore, USA), and the filtered supernatant was used to inoculate intestinal porcine epithelial cells, clone DQ (IPEC-DQ) cells (MOI = 1). In the pretreatment experiment, IPEC-DQ cells were pretreated with *B. subtilis* over 12 h, after which the supernatant was discarded. The cells were then washed 3 times with PBS to remove any residual *B. subtilis* before inoculation at an MOI of 1. Both experimental groups of IPEC-DQ cells were maintained in 2% DMEM supplemented with 50 μg/ml of gentamicin to prevent contamination. The infection rate was then evaluated by measuring GFP expression after 36 h of viral incubation, and the antiviral effect was determined by quantifying the reduction in fluorescence intensity. The viral inhibition rate was calculated using the following formula: Viral inhibition rate (%) = [(Average fluorescence intensity in the virus-infected group − Average fluorescence intensity in the probiotic-protected group)/Average fluorescence intensity in the virus-infected group] × 100%.

To further assess the antiviral activity of the candidate *B. subtilis* strains (NS1, NS3, NS7, NS10, NS11, NS12, and NS13) against PEDV, PRV, PRRSV, and SIV, each strain was incubated with the respective virus at 37 °C for 1 h prior to inoculation into the corresponding cell lines (Vero E6 for PEDV, PK-15 for PRV, Marc145 for PRRSV, and A549 for SIV) and incubated for 24 h, and the transcriptional levels of the viral genes PEDV N, PRV gB, PRRSV N, and SIV HA were quantified using RT-qPCR, with the corresponding primers listed in Table S1. Viral proteins were detected by Western blot analysis, and viral loads in the supernatants were quantified using plaque formation assays.

### In vivo protection studies of *B. subtilis* NS12

For the PRRSV challenge experiment, 20 four-week-old pigs were randomly allocated to 4 groups: mock control (group I), PRRSV infection (group II), *B. subtilis* 168 treatment (group III), and *B. subtilis* NS12 treatment (group IV). Pigs in groups III and IV received intranasal inoculations of *B. subtilis* strains 168 and NS12, respectively, on days 1 and 3, while pigs in groups I and II were administered an equivalent volume of LB medium. Twenty-four hours after the second intranasal inoculation, pigs in groups II, III, and IV were challenged with 2 ml of PRRSV (4 × 10^5^ 50% tissue culture infective doses/ml), which was administered both intranasally and intramuscularly (1 ml for each), and pigs in group I were given 2 ml of DMEM as a control. PRRSV replicates rapidly within the nasal mucosa, with a significant release of progeny viruses occurring approximately 12 h post-infection [61]. Given that *B. subtilis* NS12, as an environmental strain, has a limited capacity to rapidly colonize the nasal mucosa and effectively secrete antiviral metabolites, a single intranasal administration may not provide sufficient antiviral protection. Therefore, 12 h after the PRRSV challenge, pigs in groups III and IV received a second intranasal dose of *B. subtilis* strains NS12 or 168, respectively, in accordance with the initial experimental protocol. Clinical symptoms and body temperatures were monitored daily throughout the study period. All animals were euthanized 28 days post-inoculation for necropsy. Lung tissues were harvested, fixed in formalin, and subjected to histopathological examination using hematoxylin and eosin (H&E) staining to assess pathological changes, and IF staining was performed on lung tissue sections to detect PRRSV antigens.

For the challenge experiment with PEDV, 16 one-day-old piglets were randomly assigned to 4 groups: PEDV challenge (group I), *B. subtilis* 168 treatment (group II), *B. subtilis* NS12 treatment (group III), and the control (group IV). On days 1 and 3, piglets in groups II and III received intranasal administration of *B. subtilis* strains 168 or NS12 (1 × 10^9^ CFU), respectively, while piglets in groups I and IV were administered an equivalent volume of LB medium as a control. Twenty-four hours after the second intranasal administration, piglets in groups I and II were intranasally inoculated with 1 ml of PEDV (10^4^ PFU/ml), while those in group III received 1 ml of DMEM. Six hours post-infection, piglets in groups II and III received an additional intranasal dose of *B. subtilis* strain 168 or NS12, respectively, and the control groups were given an equivalent volume of LB medium orally. Subsequently, the protective effects of orally administered *B. subtilis* against PEDV infection were evaluated in the 4 groups. On days 1 and 3, piglets in the treatment groups received *B. subtilis* (2 × 10^9^ CFU) and control piglets were given LB medium. After 24 h, piglets in groups I and II were challenged with 2 ml of PEDV (10^4^ PFU/ml) via oral inoculation, whereas piglets in group III received 2 ml of DMEM. As our previous studies have shown, progeny PEDV viruses are released in large quantities starting 12 h after infection of the nasal mucosa [62]. Based on the same rationale as the PRRSV challenge protection experiment, piglets in groups II and III received an additional oral dose of *B. subtilis* strains NS12 and 168, respectively, 12 h post-PEDV infection, in accordance with the initial dosing protocol.

Throughout the experiment, piglets were artificially fed milk every 3 h to meet or exceed their nutritional requirements (National Research Council, 2012). Following PEDV inoculation, piglets were monitored daily for clinical signs of diarrhea. Severe watery diarrhea and vomiting were typically observed in group I piglets around 48 h post-infection, after which the piglets were euthanized with 100 mg/kg pentobarbital sodium for macroscopic examination. Small intestinal tissues were then collected for further analyses.

For the PRV challenge, the median lethal dose (LD₅₀) of PRV ZJ01 was determined through nasal inoculation. Subsequently, 66 BALB/c mice were divided into 6 groups of 11 mice to evaluate the protective efficacy of *B. subtilis* NS12 against PRV infection. Six groups were examined: the blank control (group I), PRV infection (group II), *B. subtilis* 168 nasal spray (group III), *B. subtilis* NS12 nasal spray (group IV), *B. subtilis* 168 pretreatment PRV (group V), and *B. subtilis* NS12 pretreatment PRV (group VI). Mice in group IV received *B. subtilis* NS12 (1 × 10^8^ CFU) via nasal administration for 5 consecutive days, while those in group III received the same dose of *B. subtilis* 168 and mice in groups I and II were given an equivalent volume of LB medium as a control. Two days after the administration of *B. subtilis*, mice were lightly anesthetized with isoflurane and intranasally challenged with 5 LD_50_ of PRV (groups II, III, and IV), with group I receiving an equivalent volume of DMEM as a negative control. Mice in groups IV and V were given LB medium daily via nasal administration throughout the experiment. On the second day, mice were administered an intranasal dose of PRV that was preincubated with *B. subtilis* strains 168 and NS12 at 37 °C for 1 h.

In a follow-up study to examine the antiviral activities of piceatannol in vivo, 55 BALB/c mice were divided into 5 groups: a blank control (group I), PRV infection (group II), low-dose piceatannol (group III), high-dose piceatannol (group IV), and piceatannol pretreatment virus (group V). Mice in the piceatannol treatment groups received either low or high doses of piceatannol via nasal spray over 5 consecutive days (groups III and IV, respectively), while groups I and II received an equivalent volume of dimethyl sulfoxide (DMSO) as a solvent control. On the second day post-spraying, groups II, III, and IV were intranasally challenged with PRV, whereas group I received the same volume of DMEM. Throughout the experiment, mice in group V received DMSO nasal sprays daily and were administered piceatannol-pretreated PRV. At 60 h post-infection, 5 mice from each group were randomly selected, euthanized via cervical dislocation, and necropsied to observe tissue lesions in the brain, lungs, and liver. Viral loads were quantified by RT-qPCR. Survival curves were generated for the remaining 6 mice per group, which were monitored daily.

### Histopathology and IF staining

Collected tissues were fixed in 4% paraformaldehyde and embedded in paraffin for tissue slice preparation (4 μm). Tissue slices were then H&E-stained, and histopathological analysis was performed using a microscope. To assess the distribution of PEDV and PRRSV in the small intestine and lungs, tissue sections were subjected to IF staining using primary antibodies against the viral proteins. IF was performed as previously described. Briefly, tissue sections were fixed and permeabilized with 0.1% Triton X-100, followed by blocking with 5% bovine serum albumin. Sections were then incubated overnight at 4 °C with primary antibodies (anti-PEDV N or anti-PRRSV N mouse monoclonal antibodies) and with fluorescent secondary antibodies at room temperature for 1 h. Incubation was performed in a wet box to prevent drying. Sections were then visualized by confocal laser scanning microscopy using an LSM 710 IF microscope (Zeiss, Germany). Images were analyzed using ZEN 2012 (version 8.0), and fluorescence quantification was performed using ImageJ (version 1.53).

### Viral titer and Western blot analysis

The titers of PRRSV, PEDV, PRV, and SIV were determined using plaque assays. For each virus, the following specific host cell lines were used: Vero E6 for PEDV, PK-15 for PRV, MARC-145 for PRRSV, and MDCK for SIV. Confluent monolayers of the respective host cells, cultured in 6-well tissue culture plates, were infected with 500 μl of 10-fold serial dilutions of supernatant samples. The cells were incubated for 1 h at 37 °C, followed by overlaying with 0.7% agarose in DMEM supplemented with 2% FBS. The plates were further incubated at 37 °C for 3 days, and plaques were visualized by staining with crystal violet. Western blotting was performed to determine the quantity of intracellular viral proteins. Anti-PEDV N, anti-PRRSV N, anti-PRV gB, and anti-SIV HA mouse monoclonal antibodies were used as primary antibodies. Western blotting was performed in triplicate, and representative samples were shown. Band density was measured using ImageJ software and normalized to glyceraldehyde-3-phosphate dehydrogenase (GAPDH) expression.

### Identification of antiviral *B. subtilis* NS12 components

To identify the antiviral components of *B. subtilis* NS12, the culture was centrifuged at 8,000*g* for 10 min to separate the supernatant from the vegetative cells and filtered through a 0.22-μm membrane to remove residual bacterial cells. Vegetative cells were then washed twice with PBS and heat inactivated at 80 °C for 10 min. Both the supernatant and the inactivated cells were then incubated with PEDV or PRV at 37 °C for 1 h, and viral suspensions were inoculated into Vero E6 and PK15 cells at an MOI of 1. After 24 h of incubation, viral gene expression and protein levels in the cells were analyzed by RT-qPCR and Western blotting, respectively, and viral titers in the supernatant were assessed using plaque formation assays.

### Purification and characterization of bioactive lipopeptides produced by *B. subtilis* NS12

Lipopeptides from *B. subtilis* NS12 were extracted from culture supernatants using previously published methods. Briefly, bacterial cells were separated from the fermentation broth by centrifuging at 8,000*g* for 10 min and the pH of the supernatant was subsequently adjusted to 2 with HCl to precipitate the lipopeptides, which were allowed to incubate overnight at 4 °C. The resulting precipitate was collected by centrifugation at 10,000*g* for 10 min and dissolved in 3 ml methanol. The concentrated lipopeptides were obtained by rotary evaporation under reduced pressure at 60 °C and 60 rpm. Surfactants were identified using a high-resolution tandem mass spectrometry system (Triple TOF 5600+ LC/MS/MS). Chromatographic conditions included an injection volume of 4 μl, a column temperature of 35 °C, a detection wavelength of 210 nm, and a flow rate of 0.84 ml/min. Mobile phases comprised acetonitrile (phase A) and 0.1% formic acid in water (phase B), with gradient elution of 0 to 5 min with 90% to 40% B, 5 to 15 min with 40% to 5% B, 15 to 25 min with 5% B, and 25 to 32 min with 90% B. The information-dependent acquisition method was used for MS analysis, with an *m*/*z* range of 100 to 1,500 and electrospray ionization source in positive mode.

### Nontargeted metabolomics analysis of the culture supernatants of *B. subtilis* NS12

Culture supernatant of *B. subtilis* NS12 was collected and subjected to nontargeted metabolomic analysis at Luming Biotech (Shanghai, China), with supernatant from *B. subtilis* 168 serving as a negative control. Six fresh colonies from each strain were selected and inoculated into 2 ml of LB liquid medium, followed by incubation at 37 °C for 14 h. After centrifugation and filtration through a 0.22-μm membrane, supernatant was mixed with 600 μl of culture and 20 μl of L-2-chlorophenylalanine (0.06 mg/ml in methanol) before lyophilization. The dried sample was then reconstituted in 600 μl of methanol–acetonitrile (2:1, v/v), vortexed for 30 s, and subjected to ultrasonic extraction in an ice-water bath for 10 min. After centrifugation, 150 μl of the supernatant was transferred to a glass derivatization vial and dried and the sample was treated with 80 μl of pyridine containing 15 mg/ml hydroxylamine. The supernatant was then vortexed and incubated at 37 °C for 90 min to facilitate oximation.

Subsequently, 50 μl of *N*,*O*-bis(trimethylsilyl) trifluoroacetamide was added to the vial with 1% trimethylchlorosilane, 20 μl n-hexane, and 10 μl of 10 internal standards (C8–C24 fatty acids in chloroform), and the mixture was incubated at 70 °C for 60 min. Samples were then cooled to room temperature over 30 min and analyzed by gas chromatography–MS on a DB-5MS capillary column with helium as the carrier gas supplied via splitless injection at 260 °C at 1.0 ml/min with the temperature ranging from 60 to 305 °C. MS was performed in the electron impact mode with a mass scan range of *m*/*z* 50 to 500. Metabolites were identified by matching the *m*/*z* values and molecular formulas using databases such as HMDB (http://www.hmdb.ca), Mass Bank (http://www.massbank.jp), and METLIN Metabolite (http://metlin.scripps.edu). Figure S15 provides the retention times, exact masses, MS/MS fragmentation spectra, and chromatograms that verify the presence of piceatannol, resveratrol, daidzein, atropine, α-tocopherol, biochanin A, and benzoic acid in the NS12 culture filtrate. Complementary high-performance liquid chromatography with photodiode-array detection (HPLC-DAD) quantification showed that, after 12 h of incubation, resveratrol and piceatannol reached concentrations of 385.34 ± 35.0 μg/l and 168.10 ± 4.12 μg/l, corresponding to 3.8-fold and 2.0-fold increases, respectively, relative to the *B. subtilis* 168 control (Fig. S15).

### Identification of antiviral metabolites in secretions from *B. subtilis* NS12

Based on the results of untargeted metabolomics and the relevant literature, 8 metabolites with potential antiviral activities were identified: daidzein, atropine, benzoic acid, α-tocopherol, resveratrol, desaminotyrosine, biochanin A, and piceatannol [51,63–66]. The antiviral activities of the selected compounds were assessed using plaque inhibition assays, with optimal concentrations determined based on CCK-8 assay results and the existing literature. Vero E6 or PK15 cells were cultured until approximately 85% confluence, and then a suspension containing 1 × 10^5^ PFU/ml of PEDV or PRV was incubated with the selected metabolites at 37 °C for 1 h. These mixtures were then added to the cells and incubated for 30 min at 4 °C to facilitate viral adsorption, followed by an additional 30-min incubation at 37 °C. The cell supernatant was then discarded, and the cells were washed to remove any unbound viruses. Finally, DMEM supplemented with 1% agar was added to each well. Following a 72-h incubation period at 37 °C, cells were fixed with 4% formaldehyde, the agar overlay was removed, and plaques were visualized using 0.1% crystal violet stain.

### Identification of the antiviral stage of piceatannol and analysis of its time and temperature dependencies

The antiviral activities of piceatannol against PEDV and PRV were evaluated using various treatment protocols. (a) Piceatannol pretreatment of cells: Vero E6 or PK15 cells were treated with different concentrations of piceatannol (0, 25, 50, or 100 μM) for 1 h at 37 °C in a 5% CO₂ incubator. Following treatment, the medium was replaced with blank DMEM and the cells were infected with 0.1 MOI of either PEDV or PRV. Viral replication was assessed 24 h post-infection. (b) Piceatannol pretreatment of viruses: Various concentrations of piceatannol (0, 25, 50, or 100 μM) were incubated with 0.1 MOI of PEDV or PRV at 37 °C for 1 h, after which the mixture was inoculated into Vero E6 or PK15 cells. Viral infection was evaluated after 24 h. (c) Piceatannol addition during viral attachment: Piceatannol was incubated with 0.1 MOI of PEDV or PRV at 37 °C for 1 h and then added to precooled Vero E6 or PK15 cells. Cells were then maintained at 4 °C for 30 min to facilitate viral attachment, after which unbound viruses were removed using blank DMEM. Viral attachment was assessed by extracting RNA for RT-qPCR analysis of PEDV and DNA for quantitative PCR of PRV. (d) Effect of piceatannol during viral invasion: Vero E6 or PK15 cells were inoculated with 0.1 MOI of PEDV or PRV and incubated at 4 °C for 30 min. Unbound viruses were then removed, and cells were treated with various concentrations of piceatannol, followed by incubation at 37 °C for 1 h. Cells were then washed and incubated for 24 h. (e) Effect of piceatannol during viral replication and release: Vero E6 or PK15 cells were inoculated with 0.1 MOI of PEDV or PRV and incubated at 37 °C for 1 h. After removing unbound viruses, cells were cultured in a 5% CO₂ incubator. Piceatannol was then added at various concentrations after 2 h, and cells further cultured for 24 h. Viral gene and protein levels were analyzed using RT-qPCR and Western blotting, respectively, and viral titers were assessed using plaque formation assays.

To investigate the antiviral mechanisms of piceatannol during viral invasion, its effects on viral attachment and invasion were assessed. Piceatannol was first incubated with PEDV or PRV at 37 °C for 1 h. Meanwhile, a 12-well plate was washed with blank DMEM and precooled at 4 °C for 30 min. Virus was then added to the precooled cells for 30 min at 4 °C, after which unbound viruses were removed. PEDV attachment was evaluated by RNA extraction and RT-qPCR, whereas PRV attachment was assessed by DNA extraction and quantitative PCR. To evaluate viral invasion, piceatannol was again incubated with PEDV or PRV at 37 °C for 1 h and viruses were added to the cells for 30 min at 4 °C, followed by the removal of unbound viruses. Cells were then incubated at 37 °C in a 5% CO₂ atmosphere for 1 h, and uninvaded viral particles were eliminated using a pH 3 citrate buffer. PEDV invasion was analyzed via RNA extraction and RT-qPCR, while PRV invasion was assessed through DNA extraction and quantitative PCR.

To evaluate the effects of incubation time and temperature on the antiviral activity of piceatannol, PEDV or PRV was incubated with piceatannol for varying durations (0, 10, 20, 40, and 60 min) at 37 °C, and at 4 °C or 37 °C for 1 h. Antiviral effects were determined using plaque reduction assays.

### Assessment of piceatannol-mediated viral nucleic acid exposure

For the viral RNA exposure assay, the impact of pterostilbene on viral RNA integrity was assessed by incubating 1 × 10^4^ PFU of PEDV with pterostilbene at 37 °C for 1 h, with Triton X-100 included as a positive control to facilitate viral membrane disruption. After the incubation, 1 μl of ribonuclease (RNase) A was added to the mixture, followed by a 1-h incubation at 37 °C to enzymatically degrade the exposed viral RNA. The reaction was subsequently neutralized by adding QVL Lysis buffer, and total viral RNA was extracted using an OMEGA Viral RNA Kit. RNA integrity and degradation were quantified using RT-qPCR to determine the extent of RNA exposure.

For the viral DNA exposure assay, 1 × 10^4^ PFU of PRV was incubated with pterostilbene at 37 °C for 1 h, using Triton X-100 as a positive control to disrupt the viral envelope and expose viral DNA. Following incubation, 10 μl of deoxyribonuclease (DNase) I was added along with 10 μl of MgCl₂, and the mixture was incubated at 37 °C for 1 h to promote the degradation of exposed DNA. DNase I activity was inhibited by heat inactivation at 80 °C for 10 min, and viral DNA was subsequently extracted using the OMEGA Viral DNA Kit, while DNA degradation was analyzed using RT-qPCR to assess the susceptibility of viral DNA to nuclease-mediated degradation.

### Membrane fusion inhibition assay

A membrane fusion inhibition assay was conducted using octadecyl rhodamine B (R18) as a fluorescent probe to assess the inhibitory effects of different compounds on viral membrane fusion. Purified PRV or PEDV was resuspended in NaCl-Hepes buffer, and the protein concentration was adjusted to 1 mg/ml using a BCA protein quantification assay. To label the viral membrane, 15 μl of a 1.4 mM R18 ethanol solution was added to the viral suspension, followed by thorough vortexing. The mixture was then incubated at 37 °C for 1 h to allow the R18 dye to integrate into the viral envelope, and unbound dye was removed using fluorescent dye removal columns to yield R18-labeled PRV or PEDV (R18-PRV or R18-PEDV).

DMSO, resveratrol, or piceatannol was then added to the R18-labeled virus preparations and incubated at 37 °C for 1 h to evaluate the potential antiviral effects. After incubation, the treated viral suspensions were added to monolayers of Vero E6 and PK-15 cells and cultured in 96-well plates. Virus was allowed to adsorb onto the cells at 4 °C for 30 min. After adsorption, unbound viral particles were removed by washing with a prechilled fusion buffer, and fusion was induced by lowering the pH to 5 using hydrochloric acid. The fluorescence signal, indicative of viral membrane fusion, was monitored continuously for 1 h at 37 °C using a fluorescence microplate reader with excitation at 560 nm and emission at 590 nm. Readings were obtained every 30 s. At the end of the assay, 0.5% Triton X-100 was added to each well to achieve complete viral membrane disruption, and the plates were incubated at 37 °C for an additional hour to measure the maximum fluorescence intensity (*F*_max_). The percentage of membrane fusion was calculated using the following equation: Fusion rate (%) = (*F*_x_ − *F*_initial_)/(*F*_max_ − *F*_initial_), where *F_x_* represents the fluorescence intensity at each time point, *F*_initial_ is the initial fluorescence before fusion induction, and *F*_max_ is the fluorescence after Triton X-100 treatment.

### Fluorescence spectroscopy analysis of piceatannol–lipid interactions

After harvesting and washing Vero E6 cells twice with PBS, cell pellets were resuspended in 10 ml of PBS, followed by the addition of 20 ml of chloroform–methanol solution (1:1, v/v). The suspension was then incubated at room temperature on a shaker for 90 min. To facilitate phase separation, 20 ml of a chloroform-water solution (1:1, v/v) was added and the mixture was further agitated for 20 min. After separation, the lower chloroform phase containing the lipids was collected using a separatory funnel. The lipid fraction was then dried by rotary evaporation under reduced pressure, and the lipids were resuspended in PBS. The suspension was then sonicated to obtain a uniform lipid solution. For piceatannol binding assays, Vero E6 cells or extracted lipids were incubated with piceatannol at 37 °C for 1 h, samples were centrifuged, and the supernatant was discarded. The cell or lipid pellet was then washed twice with PBS to remove unbound piceatannol. Methanol (1 ml) was added to dissolve piceatannol bound to the cells or lipids, followed by a 20-min incubation at 37 °C. After centrifugation, the supernatant was collected and the fluorescence intensity of piceatannol was measured using a fluorescence spectrophotometer at an excitation wavelength of 320 nm. In an additional experiment, lipid suspensions were prepared at a concentration of 1 mg/ml in PBS and piceatannol was added to the lipid suspension to a final concentration of 5 μM before subjecting the mixture to brief vortexing and incubating for 1 h at 37 °C. The fluorescence intensity of the suspension was then measured at an excitation wavelength of 320 nm to evaluate the interaction between piceatannol and lipids.

### MD simulation to reveal the role of piceatannol in viral membrane fusion

Structural acquisition and optimization were performed by downloading the structures of resveratrol and piceatannol in the structure data file format from PubChem (https://pubchem.ncbi.nlm.nih.gov). RDKit software was utilized to invoke the Chem [67]. The AllChem module leverages the EmbedMolecule function to create 3-dimensional molecular conformations through a refined distance-geometry algorithm that incorporates experimentally derived torsion-angle constraints [68]. Subsequently, the MMFF Optimize molecular module was used to optimize the small-molecule structures and energies using the MMFF94 force field [69].

MD simulations were conducted using Gromacs 2019.6 as simulation software. The initial configuration of the bilayer membrane, comprising an upper viral membrane and a lower cell membrane, was constructed using Packmol [70]. The viral membrane is composed of PC, PE, and PI at a ratio of 6:2:2 [71]. Ten molecules of resveratrol or piceatannol were then randomly introduced between 2 bilayer membranes, the small molecules were modeled using the General Amber Force Field (GAFF2), and the lipid structures were modeled using the lipid21 force field. The TIP3P water model was used to solvate the complex, create a water box, and add sodium ions to achieve system neutrality.

For the simulations, both Verlet and coarse-grained algorithms were applied under elastic simulation conditions, with the particle mesh Ewald method used to manage the electrostatic interactions. Energy minimization was performed using the steepest descent method for a maximum of 50,000 steps, with the cutoff distances for the Coulombic interactions and van der Waals forces set to 1.4 nm. The system was equilibrated under the canonical ensemble and the isothermal–isobaric ensemble at 300 K, followed by a 100-ns MD simulation. Hydrogen bonds were constrained during the MD simulations using the LINCS algorithm with an integration time step of 2 fs. The particle mesh Ewald method was applied for calculation, with a cutoff value of 1.2 nm for electrostatic and 10 Å for nonbonded interactions. The V-rescale method was used to maintain the simulation temperature at 300 K, and the Berendsen method was used to regulate the pressure to 1 bar. Canonical ensemble and isothermal–isobaric ensemble equilibrations were performed for 30 ps each. Finally, a production MD simulation of the protein–ligand complex was executed for 200 ns. Root mean square deviation was used to monitor conformational changes at local sites during the simulation (with a fluctuation cutoff of 0.2), the Rg was assessed to evaluate the structural compactness of the system, and the RMSF was used to track local conformational changes. The binding energy between the small molecules and cell membrane was calculated using Gmx_MMPBSA [72].

### Quantum chemical calculation

All data in this study were calculated using the Gaussian 16 software package and optimized at the B3LYP level of density functional theory [67]. The 6-31G (d) basis set was selected for all nonmetal atoms. Vibrational frequency analysis was performed to confirm that the minimum points had no imaginary frequencies, whereas the saddle points corresponded to the transition states, exhibiting one imaginary frequency. All energy values reported were Gibbs free energies of 298.15 K, which is relevant for assessing the stability and spontaneity of the studied systems.

### Cytotoxicity assay

The cytotoxicity of surfactin, piceatannol, and *B. subtilis* NS12 was evaluated using the CCK-8 assay. Vero E6 and PK-15 cells were seeded in 96-well plates and cultured to approximately 90% confluence overnight. Cells were then treated with surfactin (3.125 to 100 μg/ml) or piceatannol (3.125 to 100 μM) for 24 h, alongside blank and control groups, and subsequently washed with DMEM. For the *B. subtilis* assay, various CFUs of isolates (NS1, NS3, NS7, NS10, NS11, NS12, and NS13) were applied to Vero E6, PK15, and Marc145 cells and incubated for 1 h at 37 °C. Unbound bacteria were removed, fresh DMEM with 2% FBS was added, and plates were incubated for another 24 h. After incubation, cells were washed and treated with 10 μl of CCK-8 reagent per well. Following a 2-h incubation, absorbance was measured at 450 nm using a microplate reader. Cell viability was calculated as a percentage of the untreated control, which was set to 100%.

### Statistical analysis

All data are presented as mean ± SD. Analyses were performed with SPSS v17.0 (SPSS Inc., Chicago, IL, USA). For comparisons involving more than 2 groups, a one-way ANOVA was first conducted; when the overall *F* test was significant (*P* < 0.05), pairwise differences were evaluated with Fisher’s least significant difference (LSD) post hoc test. When only 2 groups were compared, an unpaired 2-tailed Student’s *t* test was used. Statistical significance was defined as *P* < 0.05, and differences were considered highly significant at *P < 0.01. Unless otherwise specified, results represent at least 3 independent biological replicates.

## Data Availability

The authors confirm that the data supporting the findings of this work are available within the paper and the Supplementary Materials or are available from the corresponding author upon reasonable request.
