# Supplementary material for: Broad-Spectrum Virus Elimination by Nasal Mucosa-Colonized Wild-Type Bacillus subtilis
Source: Research (Wash D C). 2025 Jul 17;8:0781. doi: 10.34133/research.0781 (PMC12270477; doi:10.34133/research.0781)
Supplement: Supplementary 1 — Figs. S1 to S15 Table S1 Data S1 [file research.0781.f1.zip › Supporting_information 5.28.pdf]

Supplementary Material

Broad-spectrum virus elimination by nasal mucosa colonized wild-type *Bacillus subtilis*

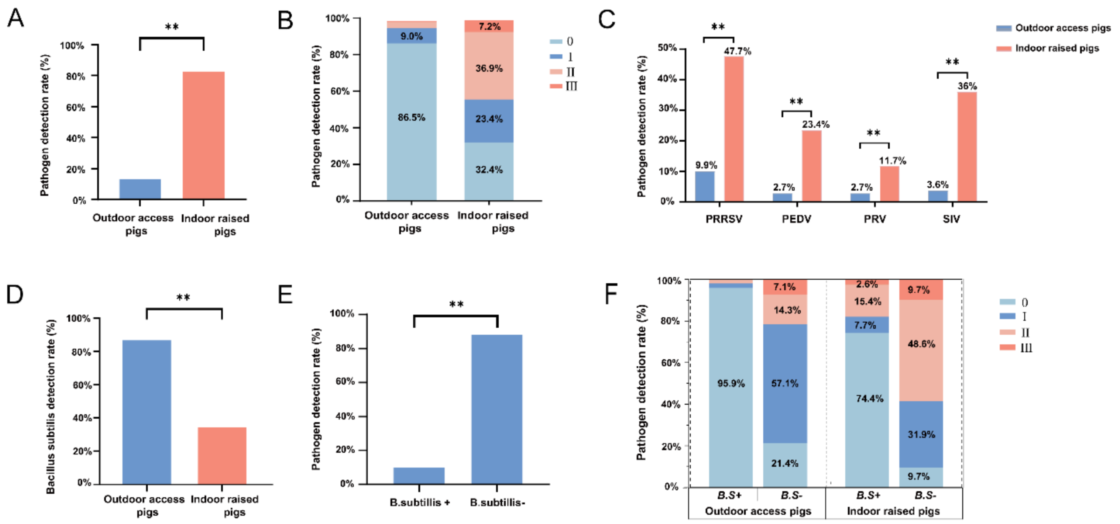

Fig. S1 Detection of respiratory viruses in nasal mucous of pigs from different rearing systems.

(A) Overall virus detection rates in pigs raised indoors versus those with outdoor access. **(B)** Proportions of viral pathogen detection in nasal swabs from pigs raised indoors and those with outdoor access. **(C)** Detection rates of various viruses in pigs raised indoors compared to those with outdoor access. **(D)** *Bacillus* detection rates in pigs raised indoors and those with outdoor access. **(E)** Detection rates of viral infections in porcine nasal mucosa with and without the presence of *Bacillus*. **(F)** Proportions of viral pathogen detection in porcine nasal mucosa with and without *Bacillus* in both indoor-raised and outdoor-access pigs. Chi-squared analysis was used to compare viral detection and *Bacillus* detection rates. *$P < 0.05$; **$P < 0.01$.

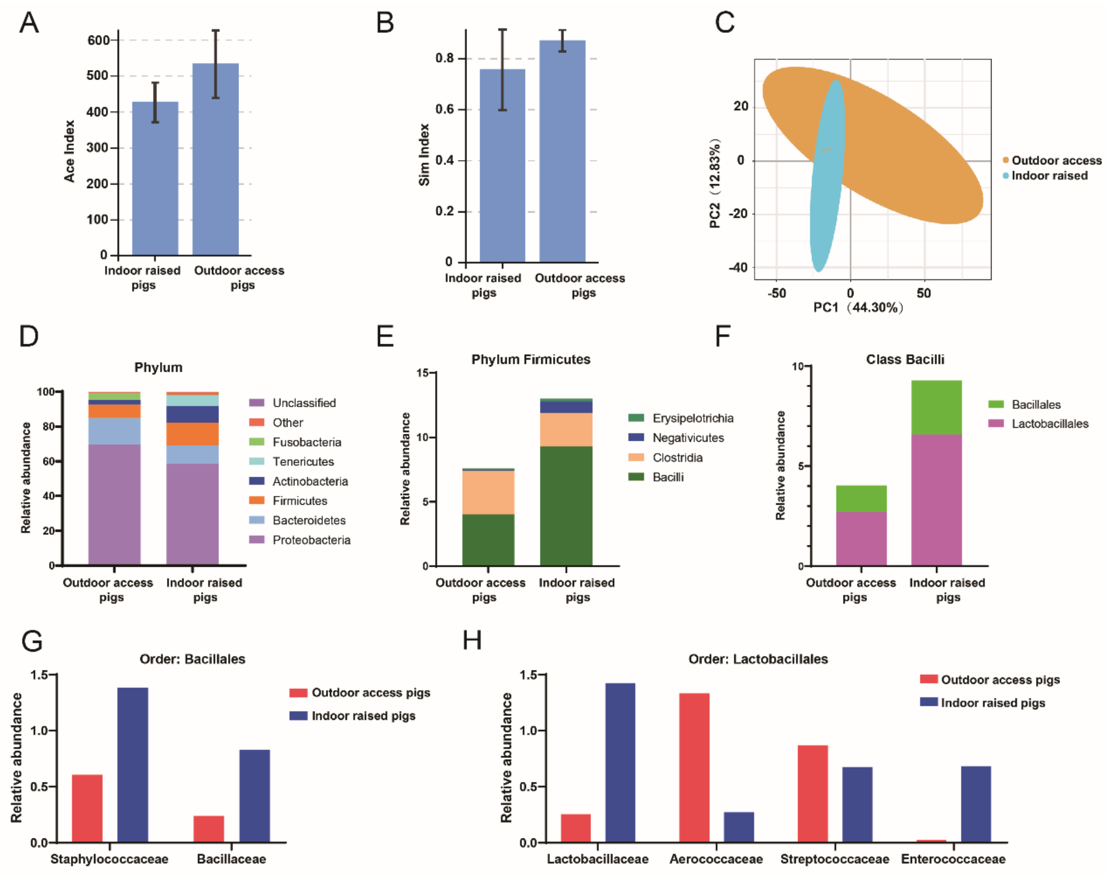

Fig. S2 Comparison of microbiota diversity and composition in the nasal mucosa of pigs from different rearing systems.

(A) Alpha diversity analysis of nasal microbiota in intensively farmed and outdoor-access pigs. **(B)** Beta diversity analysis of nasal microbiota in intensively farmed and outdoor-access pigs. **(C)** Principal component analysis (PCA) of nasal microbiota diversity in intensively farmed and outdoor-access pigs. **(D)** Composition of nasal microbiota at the phylum level in pigs from different rearing systems. **(E)** Proportions of classes within the phylum *Firmicutes*. **(F)** Proportions of orders within the class *Bacilli*. **(G)** Proportions of families within the order *Bacillales*. **(H)** Proportions of families within the order *Lactobacillales*.

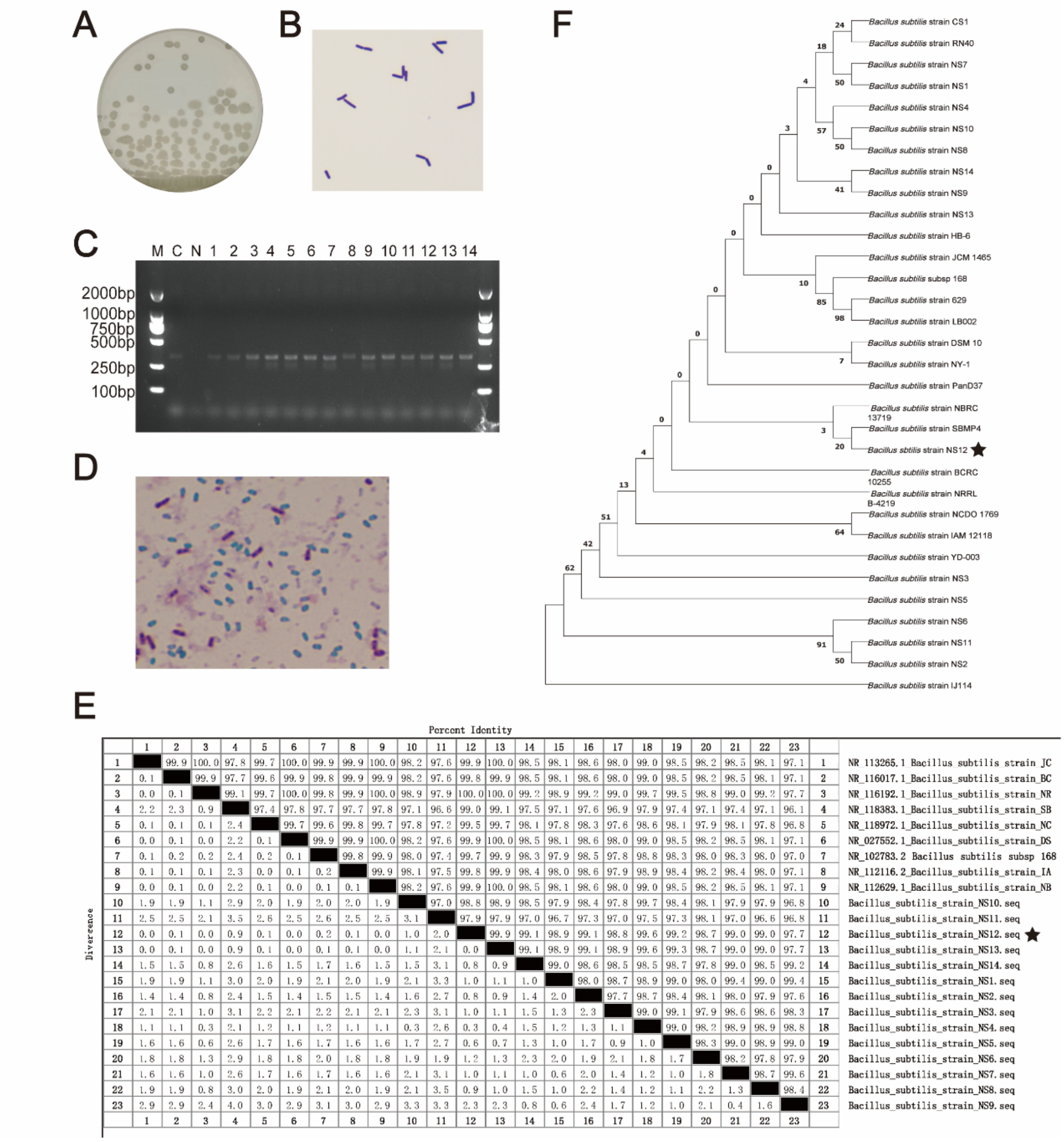

Fig. S3 Isolation and identification of *Bacillus strains* from nasal mucosa of outdoor access pigs.

(A) Colony morphology of a representative *Bacillus subtilis* strain. **(B)** Gram staining results for a representative *Bacillus subtilis* strain. **(C)** PCR amplification results of the *Bacillus* marker gene *gyrA*, with M representing the marker, C as the positive control, N as the negative control, and 1-14 corresponding to *Bacillus subtilis* isolates NS1-14. **(D)** Malachite green staining of spores from a representative *Bacillus subtilis* strain. **(E)** Phylogenetic tree depicting relationships among different *Bacillus subtilis* strains based on neighbor-joining distance analysis of 16S rRNA gene sequences. **(F)** Homology analysis of the 16S rRNA gene from various *Bacillus subtilis* strains.

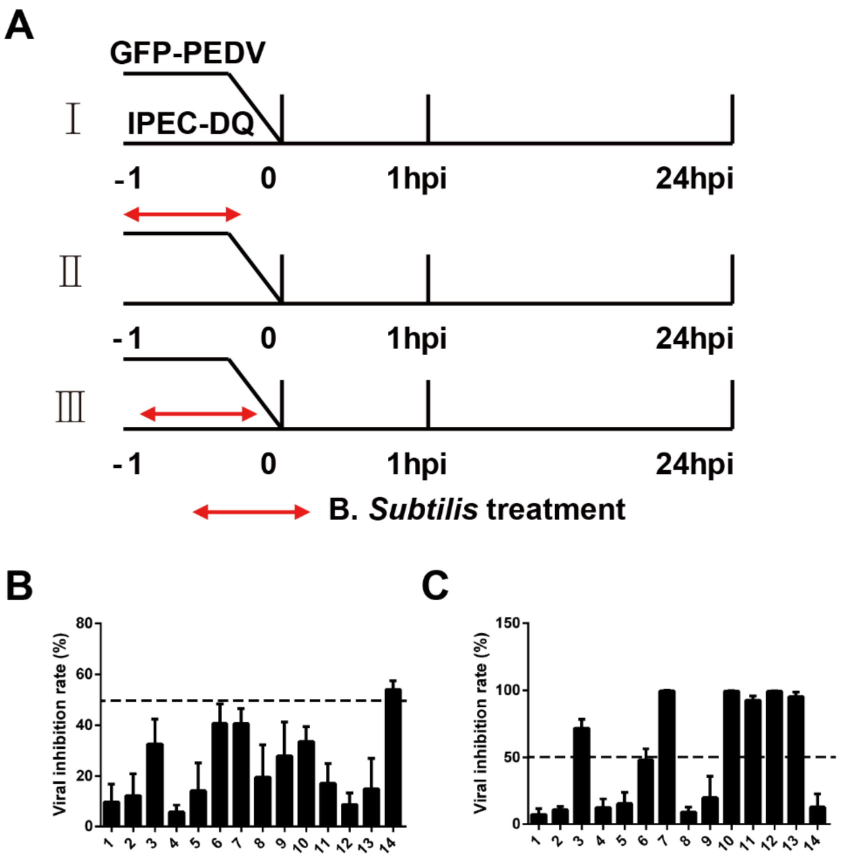

Fig. S4 *In vitro* screening of prospective *Bacillus subtilis* strains for antiviral activity against respiratory viruses.

(A) The antiviral activity of *Bacillus subtilis* isolates was assessed using PEDV expressing green fluorescent protein (GFP) via two approaches. Virus pre-treatment: *Bacillus subtilis* strains NS1–NS14 were pre-incubated with PEDV at 37 °C for 1 hour. Cell pre-treatment: Strains NS1–NS14 were pre-incubated with IPEC-DQ cells at 37 °C for 12 hours before inoculation with PEDV. After 24 hours, cellular GFP fluorescence intensity was measured using a fluorescence microplate reader. **(B)** Virus inhibition rates of different *Bacillus subtilis* strains in the cell pre-treatment approach. **(C)** Virus inhibition rates of different *Bacillus subtilis* strains in the virus pre-treatment approach. Data are presented as means ± SD, with comparisons performed by one-way ANOVA. *$P < 0.05$, **$P < 0.01$. Results are derived from at least three independent experiments.

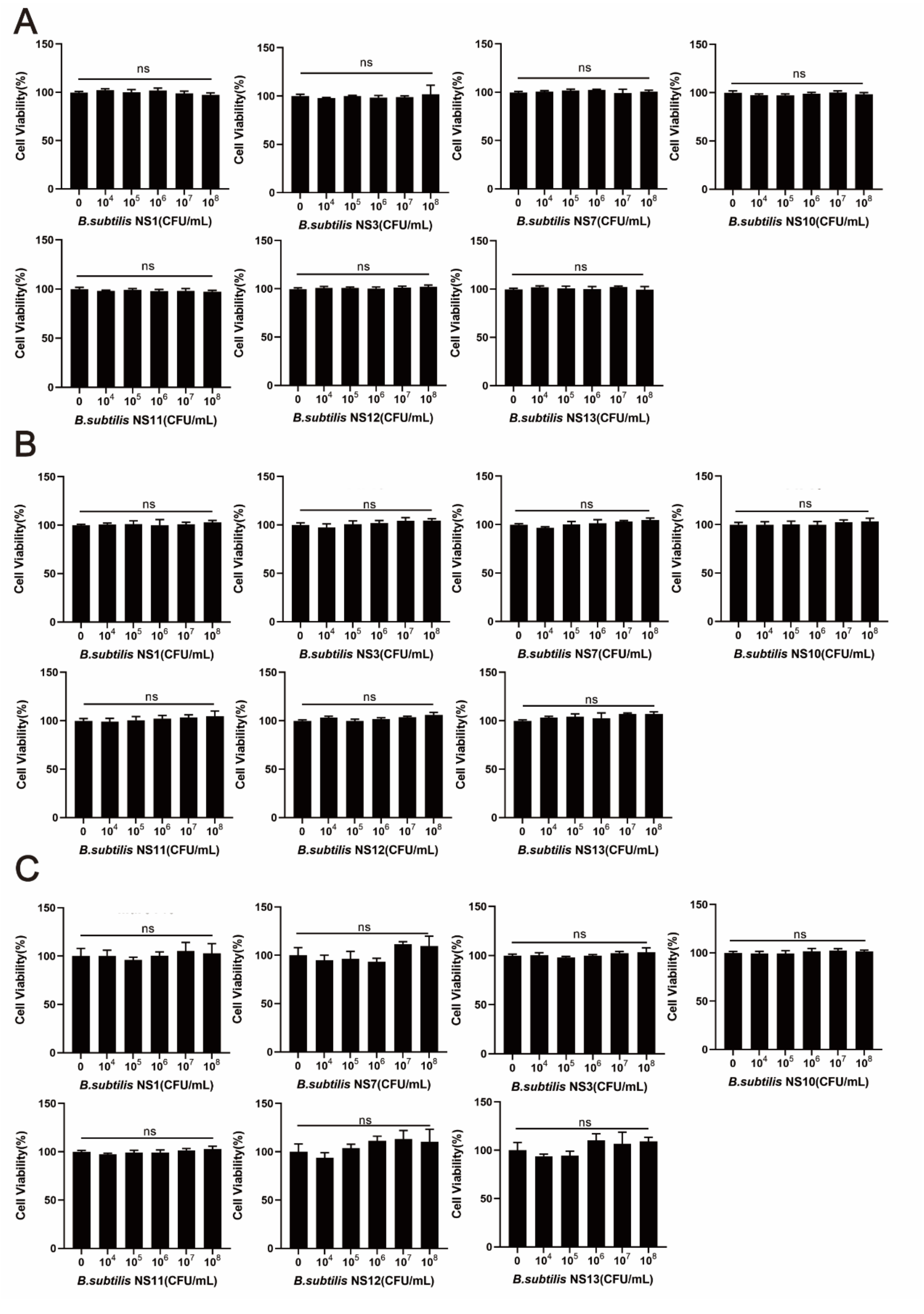

Fig. S5 The cytotoxic effects of *Bacillus subtilis* candidate on Vero E6, PK-15, and Marc145 cell lines.

(A) Cytotoxicity of candidate *Bacillus subtilis* strains on Vero E6 cells after 24 hours of incubation. **(B)** Cytotoxicity of candidate *Bacillus subtilis* strains on PK-15 cells

after 24 hours of incubation. **(C)** Cytotoxicity of candidate *Bacillus subtilis* strains on Marc145 cells after 24 hours of incubation. Data are presented as means ± SD, with comparisons performed by one-way ANOVA. *P < 0.05, **P < 0.01. Results are derived from at least three independent experiments.

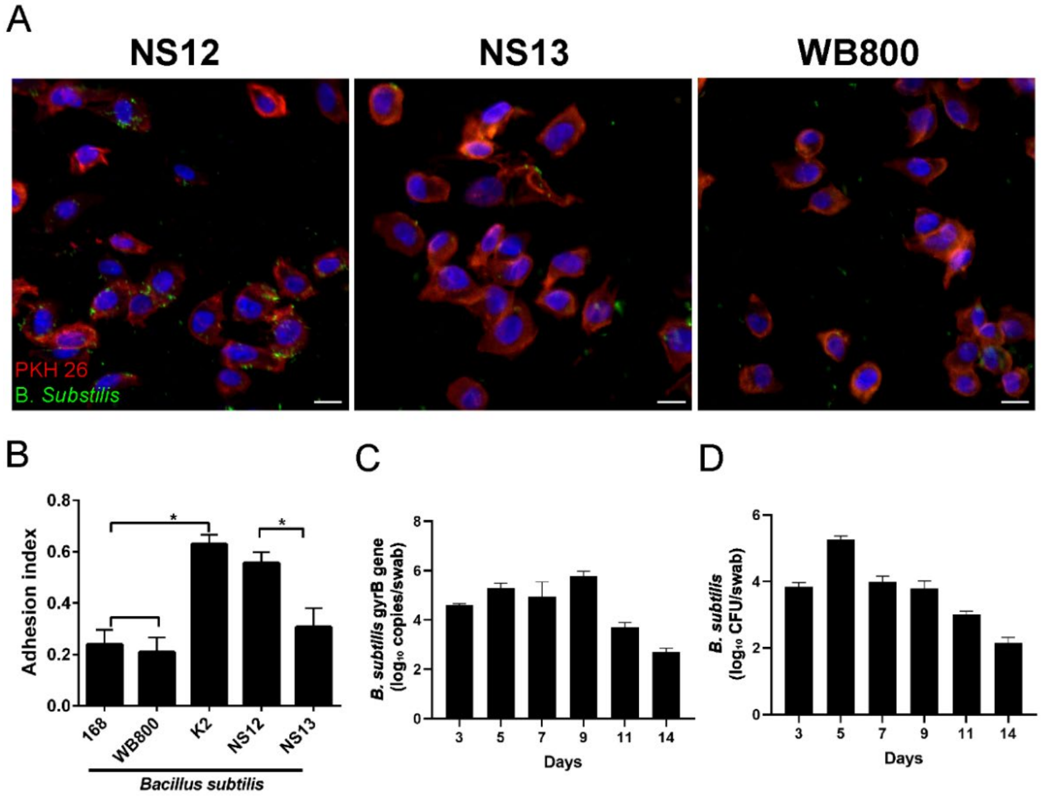

Fig. S6 Cell adhesion and nasal colonization of *Bacillus subtilis*.

(A and B) Adhesion capacity of *Bacillus subtilis* isolates NS12 and NS13 to porcine nasal epithelial cells, with *Bacillus subtilis* reference strain 168 used as a negative control. **(A)** Representative confocal fluorescence microscopy images illustrating the adhesion ability of each strain. Bacteria were stained with CFSE (green), nuclei with DAPI (blue), and cell membranes with PKH26 red membrane dye (red) (scale bar, 5 µm). **(B)** Adhesion indexes (ADI; number of bacteria per 100 cells) of each strain presented as bar graphs. **(C, D)** Nasal colonization dynamics of *Bacillus subtilis* NS12 in five-day old piglets piglets (n = 5). Nasal swabs were collected at days 3, 5, 7, 9, 11, and 14 after intranasal administration. Colonization was quantified by absolute qPCR targeting the gyrB gene **(C)** and standard plate counting **(D)**. All data are presented as means ± SD, with comparisons performed by one-way ANOVA. *P < 0.05. Results are derived from at least three independent experiments.

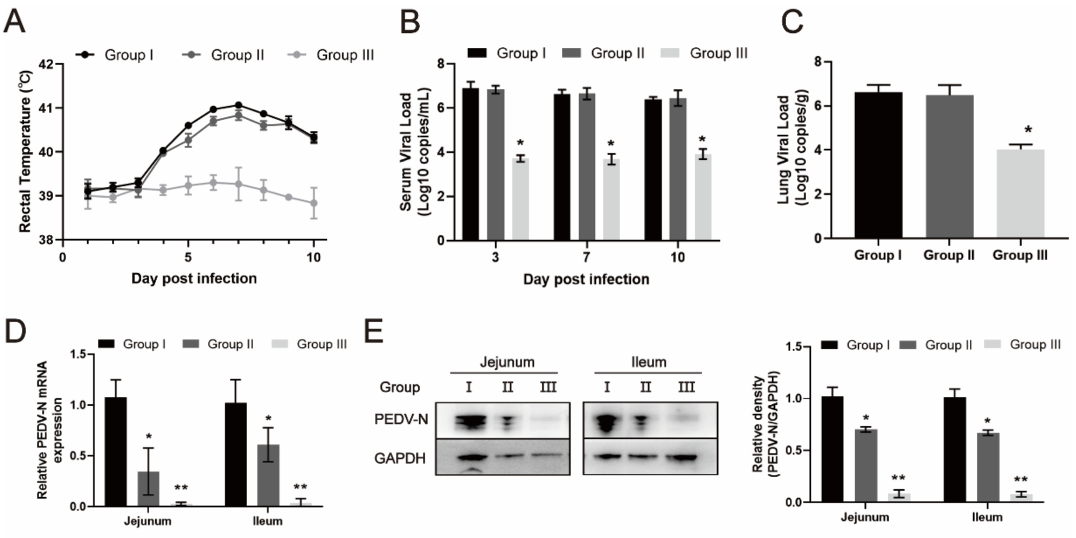

Fig. S7 Supplementary data on the protective effects of *Bacillus subtilis* NS12 against PRRSV and PEDV nasal infections.

(A -C) Evaluation of rectal temperature and serum viral load in piglets following PRRSV challenge. **(A)** Daily rectal temperature measurements for each group post-PRRSV infection. **(B)** Serum viral load quantification across different experimental groups. **(C)** Lung viral load quantification across different experimental groups. **(D and E)** Analysis of viral gene expression and protein levels in jejunum and ileum tissues following PEDV challenge. **(D)** PEDV mRNA levels determined via RT-qPCR. **(E)** PEDV protein levels assessed by Western blotting. All data are presented as mean ± SD, with comparisons performed by one-way ANOVA. *$P < 0.05$, **$P < 0.01$.

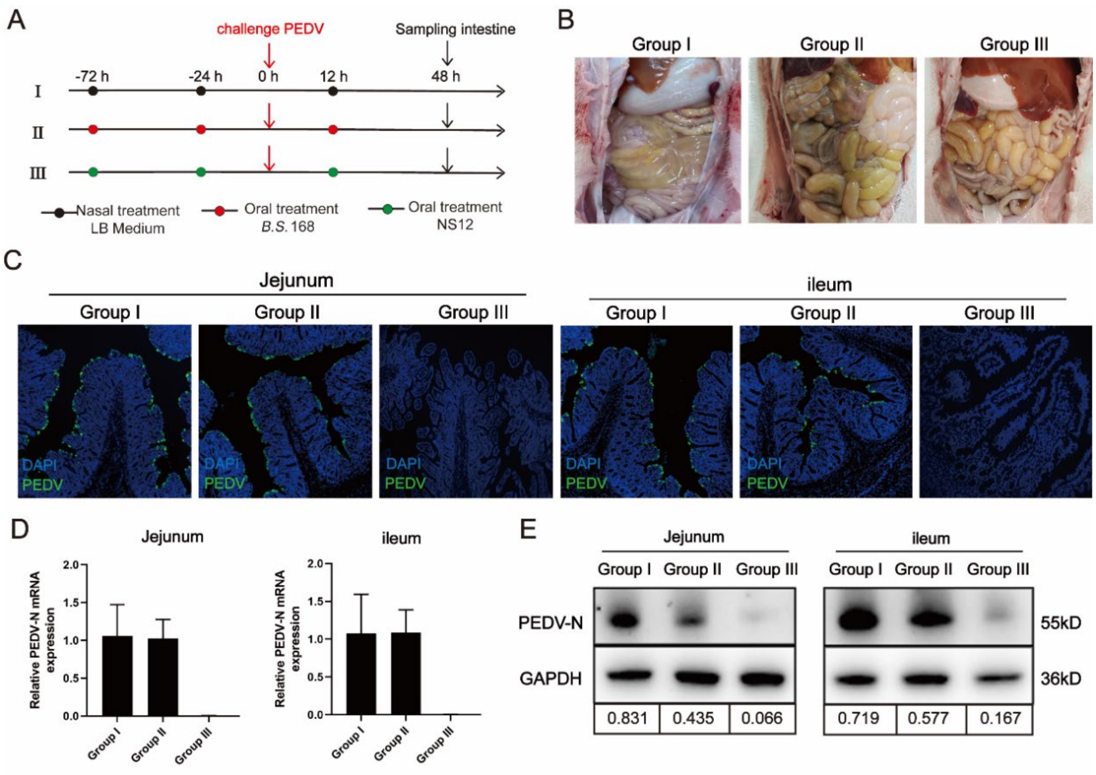

Fig. S8 Oral administration of *Bacillus subtilis* NS12 effectively protects against PEDV-induced intestinal infection.

(A) Experimental groups designed to assess the protective effects of *Bacillus subtilis* NS12 against PEDV oral infection: PEDV infection group (I), *Bacillus subtilis* 168 oral inoculation protection group (II), and *Bacillus subtilis* NS12 oral inoculation protection group (III). **(B)** Gross pathological examination of the small intestine from each group. **(C)** PEDV distribution in intestinal tissues of each group, visualized by immunofluorescence staining with anti-PEDV monoclonal antibody (green) and DAPI counterstaining for nuclei (blue) (scale bar = 50 μm). **(D and E)** Viral protein (D) and RNA (E) expression in intestinal tissues of piglets in each group (n = 4). All data are presented as mean ± SD, with comparisons performed by one-way ANOVA. *$P < 0.05$, **$P < 0.01$.

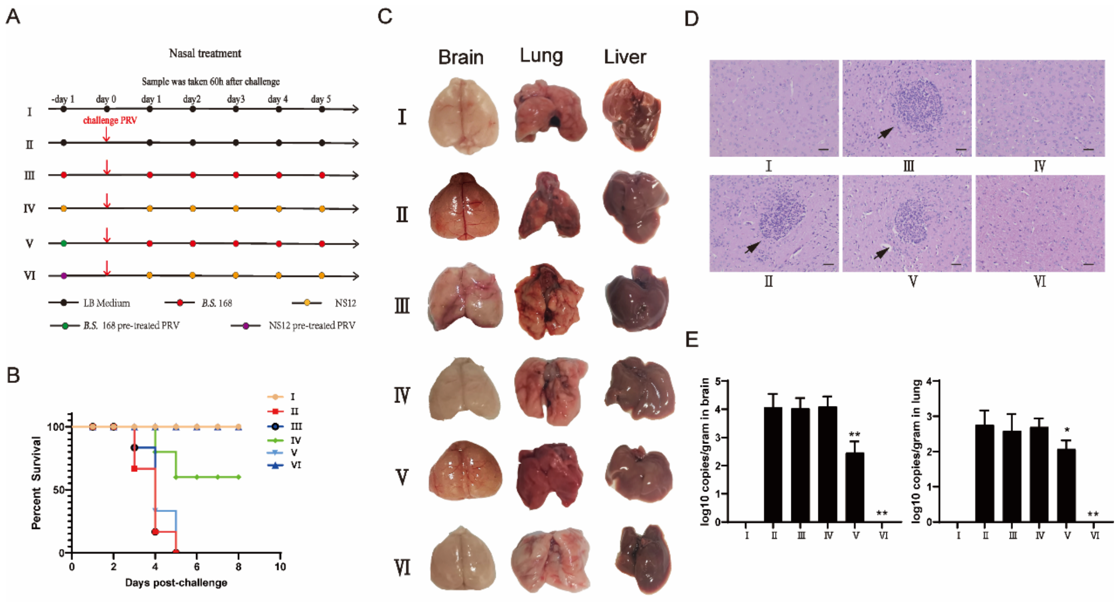

Fig. S9 Protective effect of *Bacillus subtilis* NS12 against nasal PRV infection. **(A)** The protective effect of *Bacillus subtilis* NS12 against nasal PRV infection was evaluated with the following experimental groups (n = 11 per group): blank control group (I), PRV challenge group (II), *Bacillus subtilis* 168 nasal inoculation group (III), *Bacillus subtilis* NS12 nasal inoculation group (IV), nasal infection with PRV pre-treated with *Bacillus subtilis* 168 (V), and nasal infection with PRV pre-treated with *Bacillus subtilis* NS12 (VI). **(B)** Survival curve of mice in each experimental group. **(C)** Gross pathological examination of brain, lung, and liver tissues from mice in each experimental group. **(D)** Histopathological sections of mouse brain tissues from each experimental group, with black arrows indicating inflammatory cell infiltration (scale bar = 50 μm). **(E)** Viral load in brain and lung tissues of mice in each experimental group, as detected by qPCR. All data are presented as mean ± SD, with comparisons performed by one-way ANOVA. *$P < 0.05$, **$P < 0.01$.

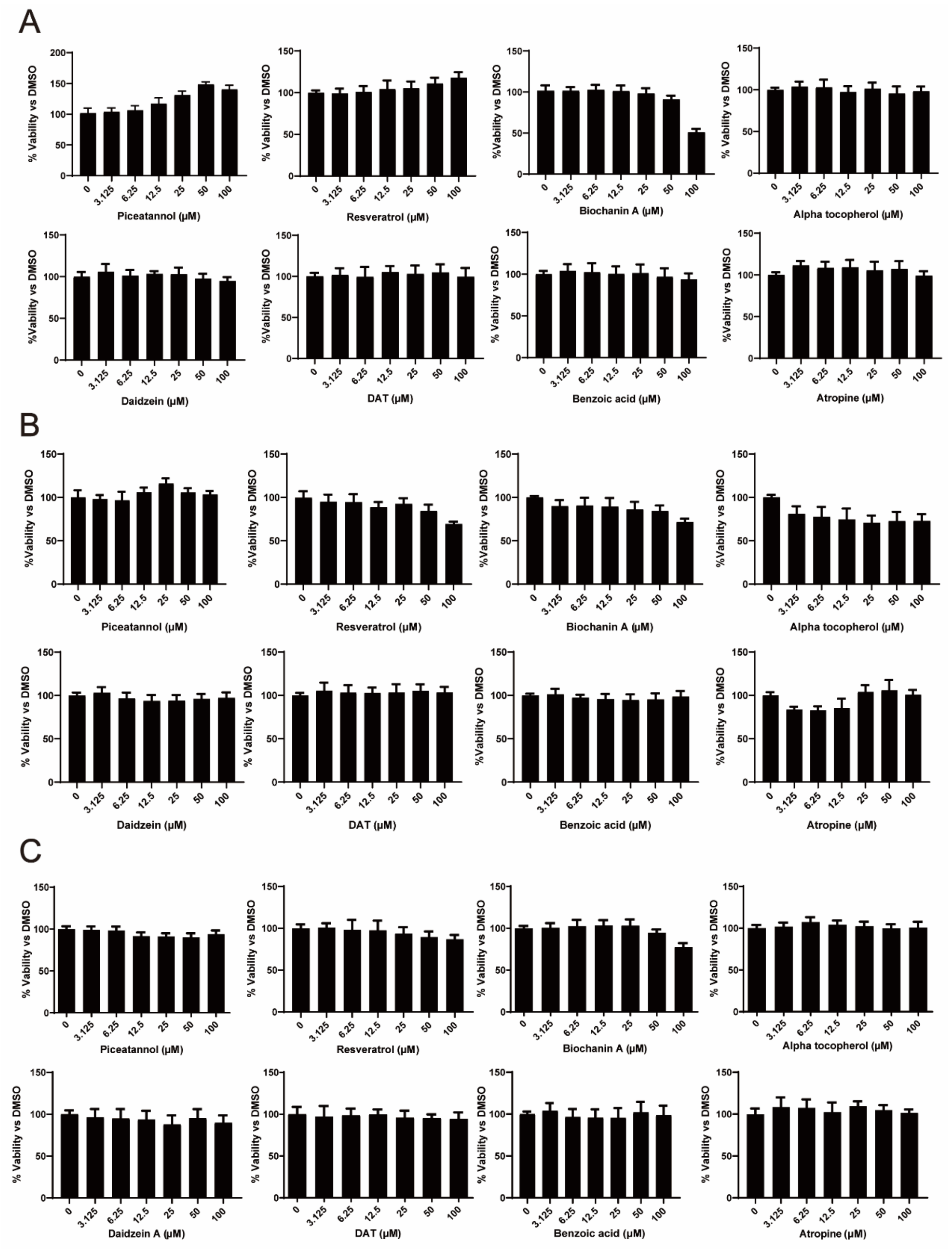

Fig. S10 Assessment of cytotoxicity of candidate small molecule compounds. **(A-C)** Cell viability of PK15 (A), Mac-145 (B) and Vero E6 (C) cells following 24 h treatment with Daidzein, Atropine, Benzoic acid, Alpha tocopherol, Resveratrol, Biochanin A, Desaminotyrosines and Piceatannol was evaluated using the CCK8 assay. Each compound was tested at a concentration gradient of 0, 3.125, 6.25, 12.5, 25, 50, and 100 μM. Data are presented as means ± SD from three independent

experiments. Statistical significance was determined using one-way ANOVA.

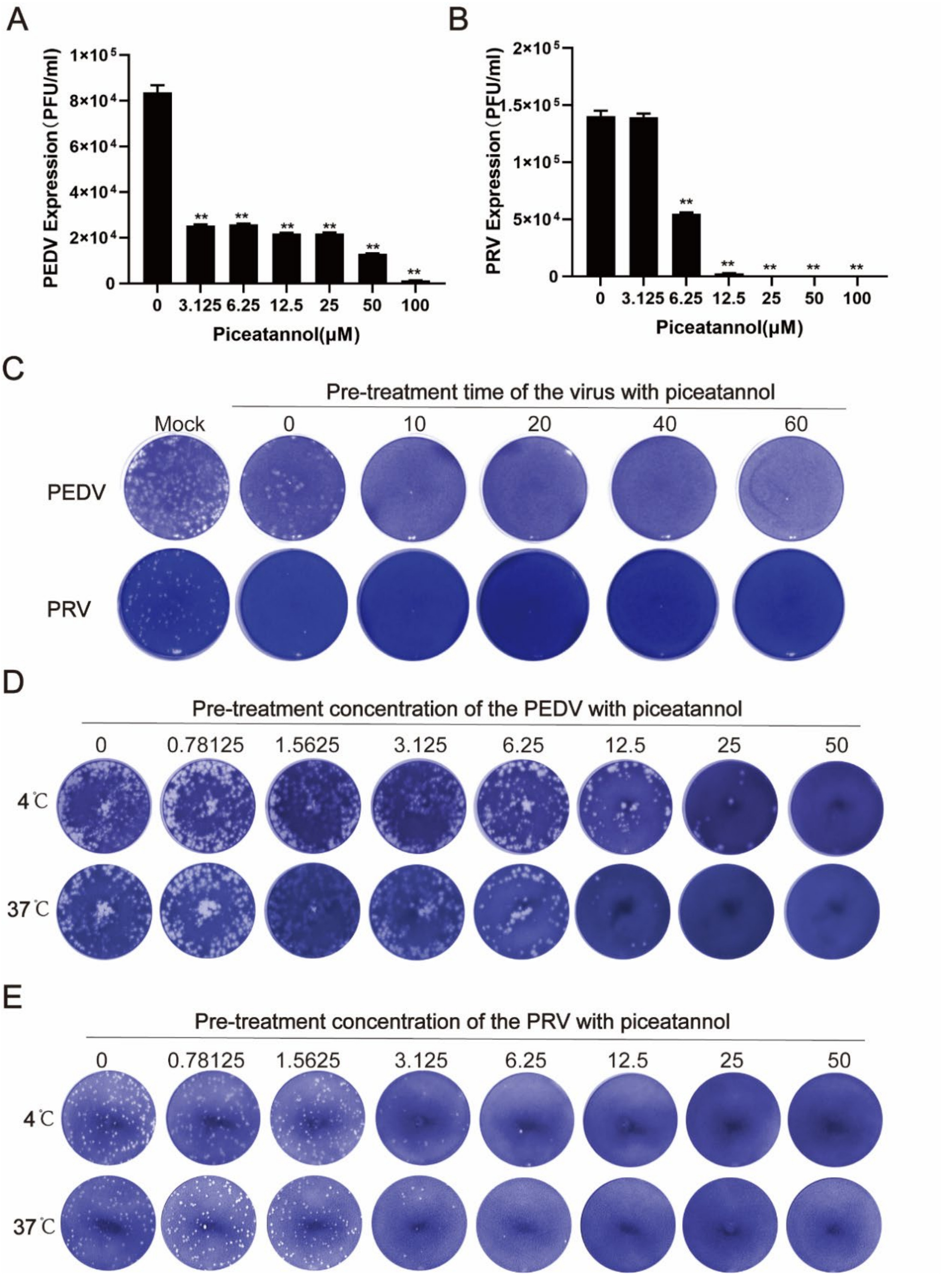

Fig. S11 Assessment of the antiviral efficacy of piceatannol treatment under varying durations and temperatures.

(A and B) Antiviral efficacy of piceatannol against PEDV (A) and PRV (B) was evaluated by plaque inhibition assays. Viral samples (1 x 10^5 PFU) were incubated with

a concentration gradient of piceatannol (3.125, 6.25, 12.5, 25, 50, and 100 μM) at 37 °C for 1 hour. **(C and D)** The impact of temperature on the antiviral activity of piceatannol was assessed by incubating a concentration gradient of piceatannolwith PEDV (C) and PRV (D) at either 4 °C or 37 °C for 1 hour, followed by plaque inhibition assays. **(E)** The effect of incubation time on the antiviral efficacy of piceatannol (80 μM) was tested by incubating with PEDV and PRV at 37 °C for 0, 10, 20, 40, and 60 minutes, with efficacy assessed by plaque inhibition assays. Data are presented as means ± SD from three independent experiments. Statistical significance was determined by one-way ANOVA; *$P < 0.05$; **$P < 0.01$.

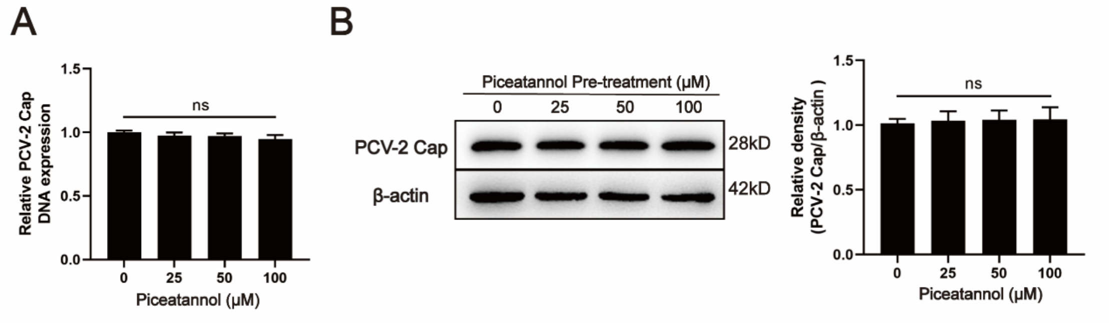

Fig. S12 Effect of piceatannol on PCV2 infection in susceptible cells.

(A and B) PCV2 was pretreated with piceatannol for 1 hour before inoculation onto PK-15 cells. After 24 hours, total nucleic acid and protein were extracted. **(A)** Viral load in cells was assessed by qPCR. **(B)** PCV2 Cap protein expression was evaluated by Western blot. All data are presented as mean ± SD, with comparisons performed by one-way ANOVA. ns, not significant.

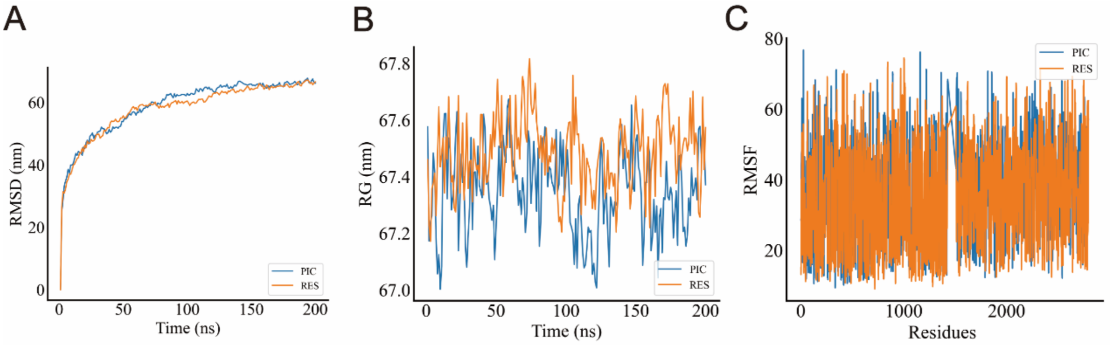

Fig.S13 Molecular dynamics stability analysis during membrane fusion.

Key metrics from molecular dynamics simulations assessing complex stability and flexibility. **(A)** Root mean square deviation (RMSD), representing the stability of the complexes throughout the simulation. **(B)** Radius of gyration (Rg), indicating the

overall compactness of the complexes. **(C)** Root mean square fluctuation (RMSF), illustrating the flexibility of individual atoms or residues within the complexes.

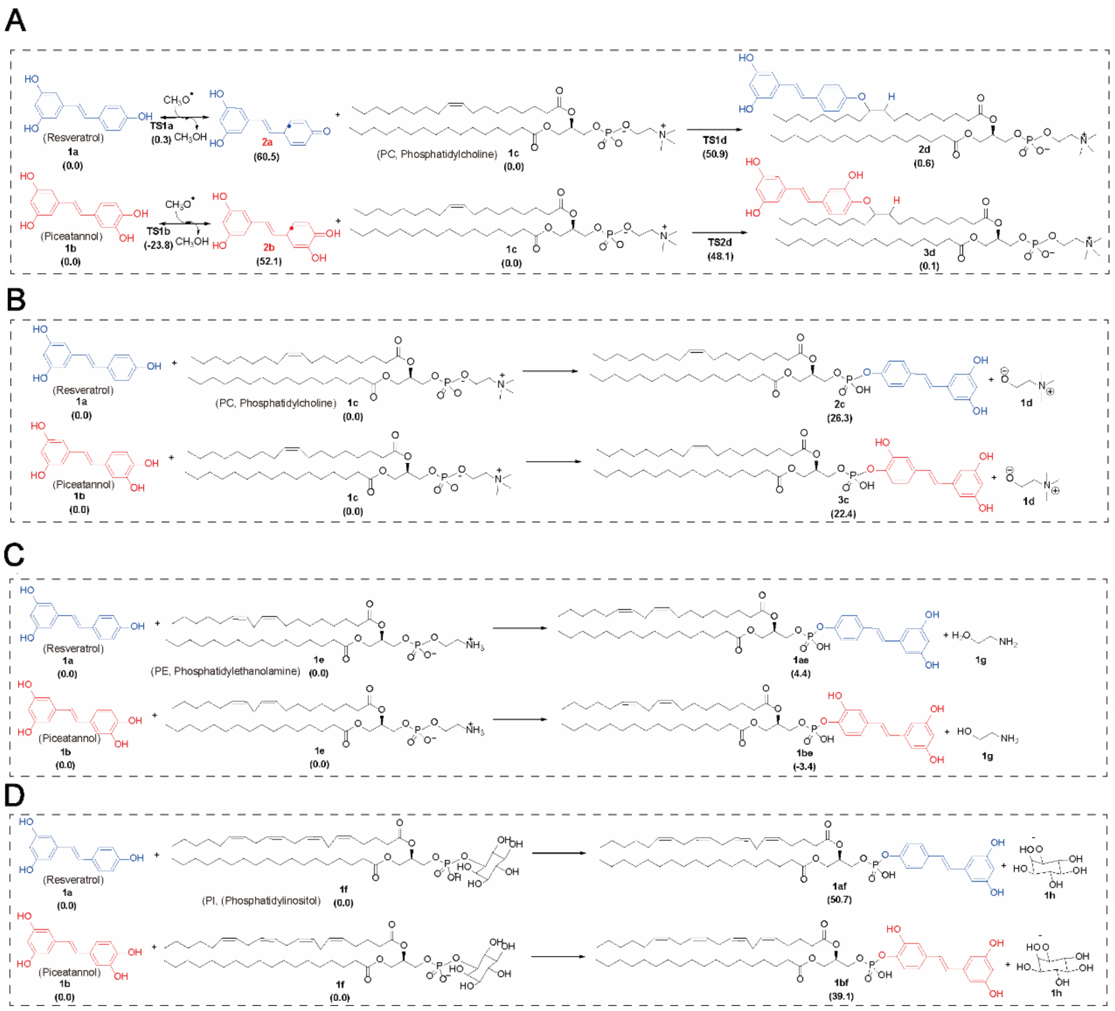

Fig. S14 Quantum chemical modeling of piceatannol-lipid interactions

(A-D) Reaction mechanism between piceatannol and PC analyzed with quantum chemical calculations, using resveratrol (lacking antiviral activity) as a control. The proposed reaction pathway includes chemical addition **(A)** and choline transfer **(B-D)** reactions. Reaction energies are presented in kcal/mol. All data are presented as means ± SD, and comparisons were performed using one-way ANOVA. *$P < 0.05$, **$P < 0.01$.

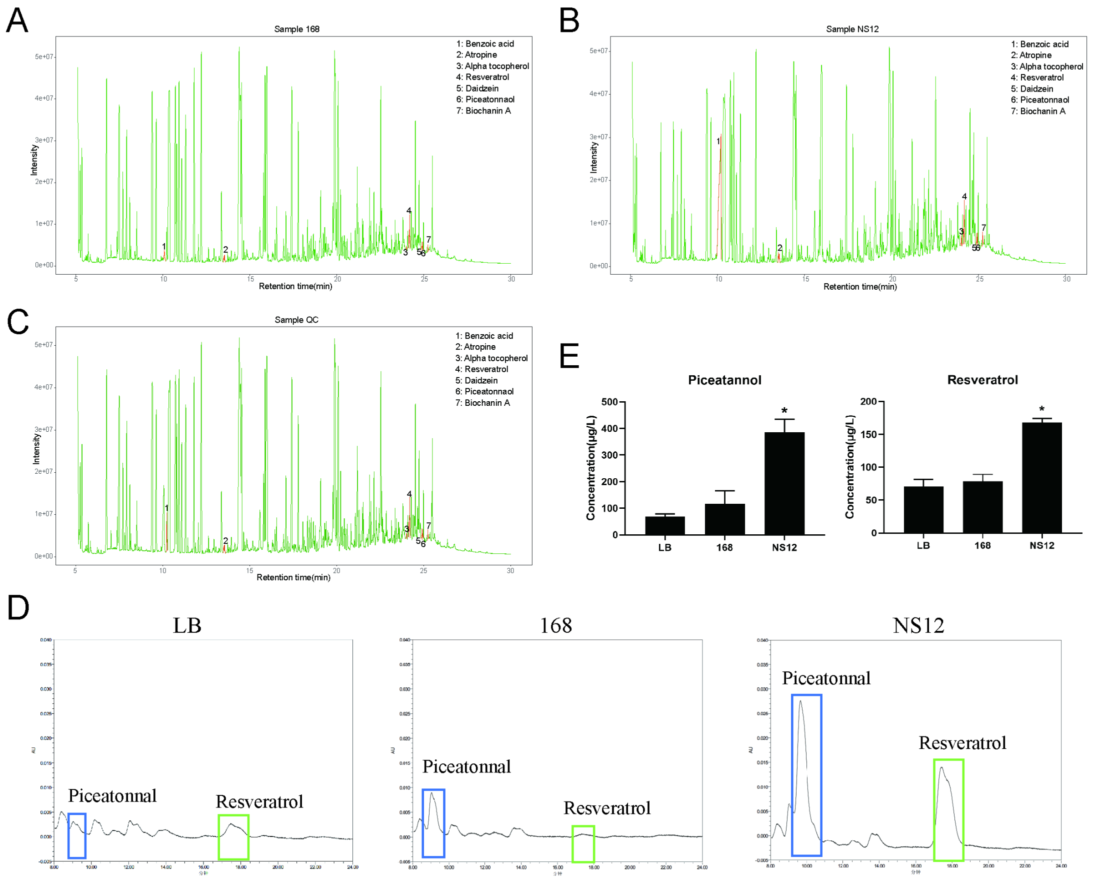

Fig.S15 Non-targeted metabolomics and HPLC analyses of culture supernatant extracts from *Bacillus subtilis* strains 168 and NS12.

(A-C) Untargeted metabolomics analysis of culture supernatants from **(A)** *B. subtilis* 168, **(B)** *B. subtilis* NS12, and **(C)** Quality control (QC) samples, revealing seven characteristic ion peaks: 1, benzoic acid; 2, atropine; 3, alpha-tocopherol; 4, resveratrol; 5, daidzein; 6, piceatannol; 7, biochanin A. **(D)** High-performance liquid chromatography (HPLC) chromatograms of metabolic extracts from LB medium (control), *B. subtilis* 168, and *B. subtilis* NS12 cultures, detected at 283 nm. Peaks corresponding to piceatannol and resveratrol are highlighted. **(E)** Quantification of piceatannol and resveratrol concentrations in metabolic extracts from LB medium, *B. subtilis* 168, and NS12 cultures. Data are shown as mean±SD (n = 3).

Table S1. Primer sequences used for RT-qPCR

Gene	Primer	Sequence (5'-3')*
**For detecting *Bacillus subtilis* in nasal cavity**		
gyrB	Forward	CTTCCACTCGCAGCTCTTCTC
	Reverse	GTRAAGTTCTCGCGCGAGT
	Probe	FAM-CCGCGTACGTGCTCCCGGACCA-BHQ1
**For isolation and identification of *Bacillus subtilis***		
gyrA	Forward	TCTGCTCGTGAACGGTGCT
	Reverse	TTTCGCCTTATTTACTTGG
16S rDNA	Forward	AGAGTTTGATCGTGGCTCA
	Reverse	TACGGTTACCTTGTTACGACTT
**For *in vitro* and *in vivo* antiviral experiments**		
GAPDH (Chlorocebus sabaeus)	Forward	ACATCATCCCTGCCTCTACTG
	Reverse	CCTGCTTCACCACCTTCTTG
GAPDH (Sus scrofa)	Forward	TCATCATCTCTGCCCCTTCT
	Reverse	GTCATGAGTCCCTCCACGAT
PEDV N	Forward	CACCTCCTGCTTCACGTACA
	Reverse	AGCTCCACGACCCTGGTTAT
PRV gb	Forward	CTTCCACTCGCAGCTCTTCTC
	Reverse	GTRAAGTTCTCGCGCGAGT
PRRSV N	Forward	ATGCCAAATAACAACGG
	Reverse	TGCTGAGGGTGATGCTGT
H1N1 HA	Forward	TCCACCTACCAGTGCTGACCAAC
	Reverse	TGCTCTTTCGGTCGGCTGCATA
**For detection of nasal virus**		
HP-PRRSV	Forward	GACGTGCCCCCAAGCTGAT
	Reverse	GGATGCCCATGTTCTGCGA
	Probe	FAM-CGTAGAACTGTGACAACAACGCTGAC-BHQ1
PEDV	Forward	CTTCCCAGCGTAGTTGAGATTGT
	Reverse	TTGCCTCTGTTGTTACTTGGAGAT
	Probe	FAM-GTTGCCATTACCACGACTCCTGCTAC-TAMRA
PRV-gb	Forward	CGGTCGTAAACGCACTATC
	Reverse	GTRAAGTTCTCGCGCGAGT
	Probe	FAM-AGAGCTTGATGTGACG-BHQ1
SIV-NP	Forward	CTTGTTCGCACCGGAAATGGAC
	Reverse	CTCCATCACCATGTCCCAAC
	Probe	FAM-GCTCTCTGATGCAGGGTTCAACTC-TAMRA
